# Supplementary material for: Quality of care of peptic ulcer disease worldwide: A systematic analysis for the global burden of disease study 1990–2019
Source: PLoS One. 2022 Aug 1;17(8):e0271284. doi: 10.1371/journal.pone.0271284 (PMC9342757; doi:10.1371/journal.pone.0271284)
Supplement: S2 Table — (DOCX) [file pone.0271284.s002.docx]

**Quality of Care of Peptic Ulcer Disease Worldwide: A Systematic Analysis for the Global Burden of Disease Study 1990-2019**

**Supplementary Table 2. Mixed-effect regression analysis to assess the validation of QCI for PUD**

| N | Dependent variable | Independent variables | | | | | Correlation with  HAQ index | |
| --- | --- | --- | --- | --- | --- | --- | --- | --- |
|  |  | Inpatient health care utilization | Outpatient health care utilization | PUD mortality | PUD prevalence | PUD mortality attributable to smoking | All causes | Cause-specific for PUD |
| 1 | QCI | ✓ | ✓ | ✓ | ✓ | ✓ | 0.4755 | 0.5041 |
| 2 | QCI | ✓ | ✓ | ✓ | 🗶 | ✓ | 0.7058 | 0.7492 |
| 3 | QCI | ✓ | ✓ | 🗶 | ✓ | ✓ | 0.2190 | 0.2874 |
| 4 | QCI | ✓ | ✓ | 🗶 | 🗶 | ✓ | 0.4877 | 0.5651 |
| 5 | QCI | 🗶 | 🗶 | ✓ | ✓ | ✓ | 0.4460 | 0.4889 |
| 6 | QCI | ✓ | ✓ | ✓ | ✓ | 🗶 | 0.4310 | 0.4707 |
| 7 | QCI | ✓ | ✓ | ✓ | 🗶 | 🗶 | 0.6493 | 0.7156 |
| 8 | QCI | ✓ | ✓ | 🗶 | ✓ | 🗶 | 0.6241 | 0.5517 |
