# Supplementary material for: Quality of care of peptic ulcer disease worldwide: A systematic analysis for the global burden of disease study 1990–2019
Source: PLoS One. 2022 Aug 1;17(8):e0271284. doi: 10.1371/journal.pone.0271284 (PMC9342757; doi:10.1371/journal.pone.0271284)
Supplement: S3 Table — (DOCX) [file pone.0271284.s003.docx]

| Location | Sex | **1990** | | | | | | **2010** | | | | | | **2019** | | | | | |
| --- | --- | --- | --- | --- | --- | --- | --- | --- | --- | --- | --- | --- | --- | --- | --- | --- | --- | --- | --- |
|  |  | **Incidence** | | **Deaths** | | **Prevalence** | | **Incidence** | | **Deaths** | | **Prevalence** | | **Incidence** | | **Deaths** | | **Prevalence** | |
|  |  | **Number** | **Rate** | **Number** | **Rate** | **Number** | **Rate** | **Number** | **Rate** | **Number** | **Rate** | **Number** | **Rate** | **Number** | **Rate** | **Number** | **Rate** | **Number** | **Rate** |
| Global | Both | 2,815,019 (2,362,080 to 3,301,983) | 63.8 (54.1 to 75.5) | 278,979 (259,455 to 301,112) | 7.4 (6.9 to 7.9) | 6,434,103 (5,405,963 to 7,627,971) | 143.4 (120.5 to 170.2) | 3,108,874 (2,637,541 to 3,583,283) | 46 (39.4 to 52.9) | 235,917 (221,136 to 255,516) | 3.8 (3.6 to 4.1) | 7,037,066 (6,016,784 to 8,236,576) | 103.1 (88.4 to 120.4) | 3,591,469 (3,031,288 to 4,217,645) | 44.3 (37.3 to 51.9) | 236,139 (216,762 to 261,413) | 3 (2.7 to 3.3) | 8,090,476 (6,794,576 to 9,584,000) | 99.4 (83.9 to 117.5) |
|  | Female | 1,214,970 (1,022,592 to 1,422,739) | 52.7 (44.5 to 62.1) | 114,045 (99,995 to 128,750) | 5.6 (4.9 to 6.2) | 2,793,426 (2,349,299 to 3,321,990) | 119.3 (100.6 to 141) | 1,458,850 (1,244,415 to 1,679,335) | 41.9 (35.8 to 48.3) | 103,393 (93,798 to 112,583) | 3.1 (2.8 to 3.4) | 3,326,879 (2,843,957 to 3,893,926) | 95 (81.2 to 111.2) | 1,731,035 (1,459,450 to 2,025,572) | 41.5 (34.9 to 48.4) | 108,617 (96,021 to 120,954) | 2.5 (2.2 to 2.8) | 3,918,557 (3,293,571 to 4,644,263) | 94.2 (79.1 to 111.9) |
|  | Male | 1,600,049 (1,337,828 to 1,890,350) | 75.6 (64.1 to 90) | 164,934 (146,881 to 180,423) | 9.6 (8.6 to 10.4) | 3,640,677 (3,024,937 to 4,345,090) | 168.4 (141 to 201.3) | 1,650,024 (1,402,835 to 1,904,664) | 50.3 (43.2 to 58.1) | 132,524 (124,627 to 145,866) | 4.7 (4.4 to 5.2) | 3,710,186 (3,163,361 to 4,354,273) | 111.6 (95.4 to 130.7) | 1,860,435 (1,566,970 to 2,184,918) | 47.2 (39.9 to 55.1) | 127,522 (115,261 to 143,080) | 3.6 (3.2 to 4) | 4,171,919 (3,492,285 to 4,965,232) | 104.9 (88.3 to 124.1) |
| **SDI** | | | | | | | | | | | | | | | | | | | |
| High SDI | Both | 439,002 (371,562 to 516,853) | 44.4 (37.7 to 52.1) | 39,550 (36,627 to 41,144) | 3.8 (3.5 to 4) | 1,009,058 (858,167 to 1,187,537) | 102.2 (86.6 to 120.4) | 469,647 (406,390 to 539,073) | 35.2 (30.4 to 40.3) | 24,461 (21,662 to 26,861) | 1.4 (1.3 to 1.6) | 1,095,169 (952,797 to 1,258,246) | 82.6 (71.4 to 95.3) | 516,386 (436,242 to 610,538) | 34.2 (28.8 to 40) | 25,352 (21,967 to 28,013) | 1.2 (1 to 1.3) | 1,210,512 (1,034,476 to 1,425,082) | 81 (68.2 to 95.8) |
|  | Female | 211,597 (177,615 to 250,992) | 37.9 (31.7 to 44.8) | 19,727 (17,585 to 20,873) | 3 (2.7 to 3.1) | 479,446 (406,596 to 566,276) | 86.3 (72.5 to 102.4) | 222,926 (192,503 to 256,736) | 30.6 (26.4 to 35.2) | 12,460 (10,462 to 14,151) | 1.1 (1 to 1.3) | 517,241 (449,089 to 595,759) | 71.6 (62.2 to 82.5) | 246,502 (208,784 to 291,056) | 30.4 (25.5 to 35.7) | 12,966 (10,654 to 14,808) | 1 (0.8 to 1.1) | 573,402 (490,071 to 678,412) | 71.5 (60.3 to 84.6) |
|  | Male | 227,406 (193,564 to 265,604) | 51.7 (44.3 to 60.3) | 19,823 (18,779 to 20,584) | 5 (4.7 to 5.2) | 529,612 (448,944 to 625,135) | 119.6 (101.7 to 140.6) | 246,721 (214,257 to 282,647) | 40.1 (34.7 to 45.9) | 12,001 (11,137 to 12,981) | 1.8 (1.7 to 2) | 577,928 (501,267 to 665,412) | 94 (81.1 to 108.8) | 269,884 (227,894 to 316,618) | 38.1 (32.1 to 44.3) | 12,386 (11,238 to 13,639) | 1.4 (1.3 to 1.6) | 637,109 (538,965 to 750,315) | 90.6 (76 to 107) |
| High-middle SDI | Both | 583,181 (486,083 to 691,574) | 51.9 (43.8 to 61.7) | 45,425 (42,411 to 48,924) | 4.6 (4.3 to 5) | 1,354,205 (1,126,371 to 1,623,343) | 119.5 (99.8 to 143.4) | 608,787 (513,629 to 712,242) | 38 (32.1 to 44.3) | 39,362 (37,215 to 41,242) | 2.5 (2.4 to 2.7) | 1,431,694 (1,202,049 to 1,695,713) | 88.9 (74.9 to 104.8) | 665,893 (557,315 to 794,748) | 36.2 (30.2 to 42.7) | 38,952 (35,625 to 41,836) | 2 (1.8 to 2.1) | 1,550,526 (1,285,171 to 1,857,861) | 84 (69.9 to 100.3) |
|  | Female | 221,562 (185,977 to 261,995) | 37.1 (31.2 to 43.9) | 16,333 (14,754 to 18,145) | 2.9 (2.6 to 3.2) | 514,942 (429,198 to 616,856) | 85.7 (71.4 to 102.4) | 257,703 (217,001 to 302,374) | 30.7 (25.8 to 35.8) | 15,511 (14,214 to 16,546) | 1.7 (1.6 to 1.8) | 610,015 (512,233 to 721,062) | 72.6 (60.6 to 85.6) | 287,016 (238,498 to 344,225) | 29.8 (24.8 to 35.2) | 16,534 (14,502 to 18,169) | 1.4 (1.2 to 1.6) | 669,098 (553,797 to 805,465) | 69.8 (57.5 to 83.7) |
|  | Male | 361,619 (301,806 to 428,972) | 68.8 (58.1 to 82.1) | 29,092 (26,676 to 31,900) | 7 (6.4 to 7.6) | 839,263 (696,790 to 1,009,819) | 157.5 (131.7 to 189.4) | 351,083 (295,121 to 409,875) | 46 (38.9 to 53.4) | 23,851 (22,716 to 25,117) | 3.6 (3.4 to 3.8) | 821,678 (691,343 to 972,350) | 106.5 (90 to 125.2) | 378,877 (318,077 to 450,056) | 42.9 (36.1 to 50.5) | 22,418 (20,228 to 24,703) | 2.7 (2.4 to 2.9) | 881,428 (730,759 to 1,054,744) | 99 (82.3 to 118) |
| Middle SDI | Both | 676,760 (561,208 to 801,379) | 54.8 (46 to 65.5) | 65,812 (59,443 to 72,958) | 7.3 (6.6 to 8) | 1,516,930 (1,252,830 to 1,823,351) | 119.1 (98.7 to 143.5) | 708,459 (595,896 to 823,446) | 34.4 (29.3 to 39.9) | 60,348 (56,452 to 66,159) | 3.8 (3.5 to 4.1) | 1,681,972 (1,412,053 to 1,988,393) | 79.7 (67.6 to 93.5) | 846,507 (707,929 to 996,725) | 33.6 (28.2 to 39.5) | 59,505 (54,130 to 66,054) | 2.8 (2.5 to 3.1) | 2,001,993 (1,652,545 to 2,392,497) | 78.2 (65.3 to 92.9) |
|  | Female | 270,534 (224,423 to 318,251) | 43.8 (36.7 to 52.4) | 26,501 (23,433 to 30,324) | 5.8 (5.1 to 6.6) | 606,905 (501,324 to 727,658) | 95.1 (78.9 to 114.1) | 334,366 (281,074 to 388,974) | 32.3 (27.4 to 37.6) | 24,639 (22,674 to 26,701) | 3 (2.7 to 3.2) | 744,824 (624,746 to 884,766) | 70.2 (59.4 to 82.4) | 410,645 (342,080 to 486,724) | 32 (26.9 to 37.8) | 25,628 (22,191 to 29,030) | 2.3 (1.9 to 2.6) | 914,648 (753,623 to 1,097,590) | 70.6 (58.4 to 84.2) |
|  | Male | 406,226 (335,755 to 484,535) | 65.8 (55.3 to 79.1) | 39,310 (33,959 to 45,563) | 8.9 (7.8 to 10.2) | 910,025 (751,862 to 1,099,360) | 142.7 (117.8 to 172.9) | 374,093 (315,271 to 434,690) | 36.6 (31.3 to 42.3) | 35,709 (33,316 to 40,847) | 4.7 (4.3 to 5.3) | 937,147 (788,126 to 1,110,088) | 89.4 (75.8 to 105.2) | 435,862 (364,493 to 513,186) | 35.2 (29.5 to 41.1) | 33,877 (30,161 to 39,560) | 3.4 (3 to 3.9) | 1,087,345 (896,821 to 1,302,607) | 86 (71.8 to 102.4) |
| Low-middle SDI | Both | 813,790 (686,845 to 959,582) | 111.7 (95.1 to 133.2) | 97,951 (87,257 to 108,104) | 16.9 (15 to 18.6) | 1,860,537 (1,569,453 to 2,193,244) | 241 (202.6 to 285) | 859,489 (727,187 to 998,067) | 71.5 (61.2 to 83.7) | 81,702 (75,285 to 89,953) | 8.6 (7.8 to 9.4) | 1,898,799 (1,619,500 to 2,227,560) | 150.9 (128.5 to 176.2) | 999,590 (841,807 to 1,172,916) | 66.3 (56.1 to 78.1) | 81,311 (72,346 to 93,692) | 6.5 (5.8 to 7.5) | 2,195,504 (1,832,150 to 2,613,422) | 140.5 (117.9 to 166.2) |
|  | Female | 362,125 (305,771 to 425,438) | 99.1 (84.4 to 117.3) | 38,637 (31,427 to 46,203) | 13.9 (11.3 to 16.5) | 841,278 (709,613 to 991,256) | 214.5 (180.8 to 251.9) | 427,422 (362,649 to 496,764) | 69.7 (59.8 to 81.2) | 36,946 (32,702 to 41,290) | 7.7 (6.8 to 8.7) | 951,737 (803,063 to 1,121,496) | 148 (125.8 to 172.3) | 518,376 (436,709 to 603,646) | 67 (56.7 to 78.4) | 38,633 (32,509 to 45,637) | 6 (5 to 7) | 1,141,830 (950,456 to 1,352,278) | 142.8 (119.5 to 169.2) |
|  | Male | 451,664 (381,068 to 538,017) | 123.6 (104.9 to 147.9) | 59,313 (49,263 to 67,005) | 19.7 (16.3 to 22.2) | 1,019,259 (853,267 to 1,212,361) | 265.6 (223.1 to 317.6) | 432,067 (364,541 to 502,376) | 73.2 (62.5 to 86) | 44,756 (41,173 to 50,858) | 9.4 (8.7 to 10.6) | 947,062 (805,055 to 1,112,970) | 153.5 (130.4 to 180.4) | 481,214 (404,408 to 567,631) | 65.3 (55.5 to 77.1) | 42,678 (36,931 to 51,046) | 7.1 (6.2 to 8.5) | 1,053,675 (879,846 to 1,256,341) | 137.8 (114.9 to 163.4) |
| Low SDI | Both | 301,358 (252,750 to 350,720) | 95.4 (81.6 to 111.3) | 30,107 (25,923 to 34,410) | 12.7 (11 to 14.6) | 691,234 (585,194 to 810,240) | 201.4 (171.1 to 236) | 389,984 (331,650 to 450,982) | 70.7 (61.4 to 81.2) | 29,916 (26,465 to 33,683) | 7.8 (6.9 to 8.8) | 926,561 (792,738 to 1,077,930) | 155.3 (133.9 to 179.1) | 479,005 (399,694 to 557,057) | 66.4 (56.8 to 76.3) | 30,884 (27,174 to 35,016) | 6.2 (5.3 to 7) | 1,128,658 (952,311 to 1,334,547) | 145.3 (123.8 to 169.9) |
|  | Female | 148,793 (124,219 to 173,530) | 93.9 (80.2 to 109.3) | 12,794 (9,502 to 16,189) | 11.5 (8.5 to 14.6) | 350,036 (293,832 to 412,181) | 200.2 (170 to 234.2) | 215,917 (181,498 to 250,689) | 77.3 (66.9 to 88.9) | 13,785 (11,582 to 16,174) | 7.4 (6.1 to 8.8) | 501,868 (424,621 to 586,518) | 165 (141.5 to 190.8) | 267,893 (223,017 to 312,598) | 73.3 (62.6 to 84.6) | 14,802 (12,425 to 17,437) | 6 (4.9 to 7.2) | 618,200 (518,321 to 730,817) | 156.5 (133.2 to 182.2) |
|  | Male | 152,565 (128,047 to 178,192) | 96.6 (82.1 to 113.4) | 17,313 (13,891 to 20,419) | 13.8 (11 to 16.4) | 341,198 (287,496 to 403,683) | 201.8 (169.6 to 239.6) | 174,067 (147,652 to 201,185) | 63.5 (55.2 to 73.1) | 16,131 (13,694 to 18,900) | 8.1 (6.9 to 9.5) | 424,692 (365,061 to 494,661) | 144.6 (124.4 to 167.9) | 211,112 (175,765 to 245,718) | 58.8 (50.5 to 68.3) | 16,082 (13,653 to 18,831) | 6.2 (5.3 to 7.3) | 510,458 (430,628 to 603,764) | 133.2 (113.4 to 156.1) |
| **GBD Super-region** | | | | | | | | | | | | | | | | | | | |
| Central Europe, Eastern Europe, and Central Asia | Both | 279,492 (234,245 to 327,152) | 61.2 (51.5 to 71.5) | 18,795 (18,007 to 19,444) | 4.2 (4 to 4.3) | 670,923 (562,893 to 795,183) | 146.1 (122.5 to 173.5) | 286,041 (243,468 to 331,474) | 56.2 (47.6 to 64.9) | 20,834 (20,142 to 21,486) | 3.9 (3.7 to 4) | 690,294 (588,319 to 809,653) | 134.8 (114.6 to 157.7) | 292,509 (247,563 to 344,980) | 54.5 (45.8 to 63.5) | 20,242 (18,363 to 22,237) | 3.3 (3 to 3.7) | 696,944 (587,886 to 823,336) | 129.4 (108.6 to 152.8) |
|  | Female | 104,125 (87,596 to 122,587) | 40.8 (34.1 to 47.7) | 6,236 (5,909 to 6,493) | 2.2 (2.1 to 2.3) | 250,625 (210,201 to 298,017) | 97.8 (81.2 to 116.4) | 117,765 (99,881 to 137,315) | 41.3 (34.6 to 47.9) | 8,041 (7,584 to 8,427) | 2.4 (2.2 to 2.5) | 284,059 (242,173 to 334,163) | 99.3 (84 to 117) | 125,128 (105,297 to 147,954) | 41.6 (34.8 to 48.7) | 8,549 (7,567 to 9,624) | 2.2 (1.9 to 2.5) | 298,723 (251,291 to 354,685) | 99.5 (82.9 to 118.6) |
|  | Male | 175,368 (146,304 to 205,996) | 86.3 (72.9 to 101) | 12,560 (11,924 to 13,068) | 7.1 (6.8 to 7.4) | 420,298 (351,653 to 496,560) | 205.7 (173.5 to 241.5) | 168,276 (142,992 to 194,468) | 73.8 (62.8 to 84.9) | 12,793 (12,480 to 13,227) | 5.9 (5.8 to 6.2) | 406,235 (346,079 to 477,707) | 176.5 (151.2 to 206.4) | 167,380 (141,454 to 197,204) | 69.4 (58.5 to 80.8) | 11,693 (10,387 to 13,108) | 4.8 (4.3 to 5.4) | 398,221 (336,078 to 470,622) | 163.9 (138.4 to 193.3) |
| High-income | Both | 446,717 (377,446 to 527,551) | 39.8 (33.8 to 46.9) | 43,235 (40,053 to 44,988) | 3.6 (3.3 to 3.8) | 1,016,695 (863,279 to 1,200,431) | 90.9 (76.7 to 107.3) | 471,454 (409,293 to 543,070) | 31.7 (27.3 to 36.3) | 25,368 (22,393 to 27,869) | 1.3 (1.2 to 1.4) | 1,091,413 (947,861 to 1,258,033) | 73.9 (64 to 85.3) | 509,834 (430,639 to 603,094) | 30.7 (25.8 to 35.9) | 26,503 (22,907 to 29,384) | 1.1 (1 to 1.2) | 1,187,608 (1,012,608 to 1,404,654) | 72.4 (61.1 to 85.7) |
|  | Female | 217,070 (181,505 to 257,790) | 34 (28.5 to 40.4) | 21,418 (19,029 to 22,660) | 2.8 (2.5 to 3) | 487,820 (412,796 to 577,006) | 77.1 (64.5 to 91.8) | 225,529 (194,468 to 259,940) | 27.4 (23.5 to 31.6) | 13,015 (10,868 to 14,815) | 1 (0.9 to 1.2) | 519,709 (450,913 to 599,767) | 63.7 (55.3 to 73.7) | 246,295 (208,639 to 291,014) | 27.2 (22.9 to 32) | 13,660 (11,183 to 15,625) | 0.9 (0.7 to 1) | 569,245 (486,092 to 675,462) | 63.7 (53.6 to 76) |
|  | Male | 229,647 (194,892 to 269,076) | 46.4 (39.6 to 54.2) | 21,817 (20,614 to 22,585) | 4.8 (4.5 to 5) | 528,875 (447,853 to 625,174) | 106.2 (90.1 to 124.9) | 245,925 (213,597 to 282,581) | 36.3 (31.4 to 41.5) | 12,353 (11,428 to 13,366) | 1.6 (1.5 to 1.8) | 571,704 (495,222 to 657,907) | 84.7 (73.1 to 97.9) | 263,539 (222,345 to 311,079) | 34.4 (29 to 40.2) | 12,843 (11,614 to 14,151) | 1.3 (1.2 to 1.5) | 618,363 (524,222 to 730,453) | 81.6 (68.6 to 96.5) |
| Latin America and Caribbean | Both | 117,506 (99,386 to 137,284) | 45.3 (38.6 to 53.4) | 15,608 (14,832 to 16,219) | 7.6 (7.1 to 7.9) | 263,725 (222,801 to 311,077) | 99.2 (84.4 to 117.4) | 116,033 (100,653 to 132,542) | 24.6 (21.5 to 28.1) | 14,654 (13,509 to 15,822) | 3.5 (3.2 to 3.8) | 252,261 (218,043 to 292,279) | 52.7 (45.7 to 60.5) | 119,012 (101,458 to 138,831) | 20 (17.1 to 23.3) | 16,521 (14,739 to 18,604) | 2.9 (2.6 to 3.3) | 250,730 (211,950 to 296,891) | 41.8 (35.5 to 49.3) |
|  | Female | 48,676 (41,197 to 56,779) | 36.7 (31.1 to 43.1) | 6,320 (5,908 to 6,650) | 6.1 (5.6 to 6.4) | 109,413 (92,004 to 129,725) | 80.1 (68.1 to 94.4) | 51,304 (44,494 to 58,687) | 20.8 (18.1 to 23.8) | 6,415 (5,748 to 7,098) | 2.8 (2.5 to 3.1) | 112,010 (96,263 to 129,853) | 44.7 (38.7 to 51.5) | 53,673 (45,745 to 62,989) | 17 (14.5 to 19.8) | 7,477 (6,524 to 8,492) | 2.4 (2.1 to 2.7) | 113,572 (96,208 to 134,652) | 35.8 (30.4 to 42.3) |
|  | Male | 68,830 (58,266 to 80,371) | 54.7 (46.6 to 64.3) | 9,288 (8,765 to 9,698) | 9.3 (8.7 to 9.7) | 154,312 (130,295 to 181,648) | 119.6 (101.5 to 141.5) | 64,729 (56,182 to 73,859) | 28.9 (25.2 to 32.9) | 8,239 (7,665 to 8,923) | 4.3 (4 to 4.7) | 140,252 (120,645 to 162,550) | 61.5 (53.2 to 70.7) | 65,339 (55,896 to 75,877) | 23.4 (20 to 27.1) | 9,045 (8,030 to 10,239) | 3.6 (3.1 to 4) | 137,158 (116,036 to 162,076) | 48.3 (41.1 to 56.8) |
| North Africa and Middle East | Both | 751,288 (623,616 to 901,337) | 57.4 (48 to 69.6) | 78,695 (68,803 to 92,695) | 7.9 (7 to 9.2) | 1,725,511 (1,418,426 to 2,093,484) | 129 (106.9 to 156.4) | 753,737 (634,215 to 887,814) | 35.3 (30 to 41.4) | 66,904 (62,157 to 74,843) | 3.9 (3.6 to 4.4) | 1,709,903 (1,433,608 to 2,034,373) | 78.6 (66.4 to 92.9) | 874,125 (729,213 to 1,048,764) | 33.4 (28 to 39.6) | 62,833 (55,694 to 72,237) | 2.7 (2.4 to 3.1) | 1,998,757 (1,642,989 to 2,425,509) | 75.7 (62.9 to 90.9) |
|  | Female | 267,415 (220,564 to 321,751) | 41.6 (34.4 to 50.5) | 30,032 (25,168 to 37,489) | 5.9 (4.9 to 7.3) | 606,593 (499,209 to 732,115) | 92.4 (76.6 to 111.8) | 291,375 (243,294 to 344,126) | 27 (22.7 to 31.9) | 25,229 (22,793 to 28,292) | 2.8 (2.5 to 3.1) | 655,426 (547,247 to 784,230) | 59.8 (50.4 to 70.8) | 345,654 (285,078 to 420,065) | 25.6 (21.3 to 30.6) | 24,452 (20,840 to 28,365) | 1.9 (1.6 to 2.3) | 785,552 (642,386 to 956,260) | 57.8 (47.9 to 69.9) |
|  | Male | 483,873 (400,035 to 579,935) | 73.4 (61.4 to 89.3) | 48,663 (40,031 to 59,012) | 10.3 (8.6 to 12.2) | 1,118,918 (920,618 to 1,354,060) | 165.4 (137.1 to 200.9) | 462,362 (389,788 to 543,709) | 43.8 (37.5 to 51.4) | 41,675 (38,153 to 48,600) | 5.3 (4.9 to 6.1) | 1,054,478 (885,871 to 1,253,402) | 97.6 (82.7 to 115.2) | 528,471 (442,620 to 632,702) | 41.6 (35 to 49.1) | 38,381 (32,731 to 45,664) | 3.7 (3.2 to 4.4) | 1,213,206 (1,002,140 to 1,462,616) | 94 (78.3 to 111.7) |
| South Asia | Both | 184,788 (153,494 to 214,772) | 56.7 (48.6 to 65) | 17,900 (14,874 to 21,753) | 8.1 (6.8 to 10) | 433,547 (363,910 to 512,370) | 125.2 (107.5 to 145.5) | 301,070 (250,737 to 349,709) | 52.1 (45.1 to 59.3) | 23,894 (20,353 to 28,361) | 6.7 (5.9 to 7.8) | 712,736 (600,997 to 834,581) | 115.8 (100.2 to 133.4) | 386,948 (317,449 to 452,038) | 51.7 (44.1 to 59.2) | 25,507 (21,908 to 29,473) | 5.5 (4.8 to 6.2) | 914,097 (760,724 to 1,086,367) | 115.2 (98.9 to 134.2) |
|  | Female | 94,527 (77,656 to 111,531) | 55.7 (47.6 to 64) | 6,955 (5,451 to 8,616) | 6.4 (5 to 7.9) | 225,506 (187,635 to 268,700) | 124.1 (105.9 to 144.1) | 164,273 (135,965 to 192,255) | 54.5 (47 to 62.3) | 10,214 (8,402 to 12,286) | 5.8 (4.8 to 6.9) | 395,439 (328,921 to 470,792) | 122.3 (105.1 to 141.3) | 211,513 (172,454 to 249,361) | 54.2 (46.2 to 62.4) | 11,211 (9,294 to 13,306) | 4.9 (4.1 to 5.7) | 509,009 (420,734 to 611,005) | 122.2 (103.6 to 142.7) |
|  | Male | 90,261 (75,116 to 105,005) | 57 (48.6 to 65.6) | 10,945 (8,310 to 14,628) | 9.8 (7.5 to 13.1) | 208,041 (174,753 to 245,487) | 124.8 (106.9 to 146.1) | 136,797 (114,097 to 159,483) | 49.1 (42.3 to 55.8) | 13,680 (10,956 to 17,035) | 7.6 (6.1 to 9.3) | 317,297 (268,582 to 372,915) | 107.9 (93.5 to 124.9) | 175,435 (145,244 to 205,569) | 48.5 (41.4 to 55.6) | 14,296 (11,678 to 17,109) | 6.1 (5.1 to 7.2) | 405,087 (339,460 to 484,246) | 106.9 (91.9 to 125.2) |
| Southeast Asia, East Asia, and Oceania | Both | 611,101 (505,734 to 742,232) | 61.8 (51.3 to 75.4) | 58,108 (48,827 to 71,277) | 7.6 (6.5 to 9.2) | 1,394,745 (1,141,896 to 1,702,487) | 138.7 (114.7 to 169.7) | 572,417 (478,590 to 683,071) | 35.4 (29.9 to 41.9) | 47,636 (43,288 to 54,458) | 3.7 (3.3 to 4.1) | 1,297,195 (1,084,075 to 1,556,861) | 79 (66.7 to 94.1) | 650,290 (534,948 to 789,785) | 32.8 (27.2 to 39.1) | 42,948 (36,862 to 50,139) | 2.4 (2 to 2.8) | 1,489,858 (1,219,467 to 1,820,690) | 74.4 (61.6 to 89.6) |
|  | Female | 211,960 (173,073 to 258,336) | 43.9 (36.1 to 53.8) | 21,571 (17,124 to 29,026) | 5.6 (4.4 to 7.3) | 478,515 (392,277 to 582,634) | 97.6 (80.6 to 118.9) | 214,190 (177,443 to 255,753) | 26.3 (22 to 31.3) | 17,454 (15,571 to 19,801) | 2.5 (2.3 to 2.9) | 480,735 (397,285 to 579,388) | 58.2 (48.6 to 69.7) | 249,650 (203,122 to 305,049) | 24.2 (20 to 29.4) | 16,137 (13,171 to 19,453) | 1.6 (1.3 to 2) | 568,501 (460,597 to 702,501) | 54.7 (44.9 to 66.6) |
|  | Male | 399,141 (328,913 to 482,113) | 79.6 (65.9 to 97.3) | 36,536 (28,316 to 46,751) | 10.2 (8.2 to 12.5) | 916,230 (749,627 to 1,117,105) | 178.9 (147.9 to 219) | 358,227 (300,907 to 427,138) | 44.9 (38 to 53.1) | 30,183 (26,844 to 36,268) | 5.1 (4.6 to 6) | 816,459 (682,689 to 979,374) | 99.9 (84.4 to 118.2) | 400,639 (331,722 to 484,065) | 41.8 (35 to 49.6) | 26,811 (21,712 to 33,333) | 3.4 (2.8 to 4.1) | 921,357 (755,977 to 1,120,824) | 94.3 (78.2 to 112.7) |
| Sub-Saharan Africa | Both | 137,049 (114,751 to 159,403) | 42.1 (36 to 49.1) | 20,260 (17,980 to 22,454) | 8.7 (7.6 to 9.7) | 322,787 (271,952 to 380,054) | 94.4 (80.7 to 110.7) | 176,662 (147,611 to 204,983) | 33.2 (28.4 to 38.2) | 18,847 (17,364 to 20,940) | 4.8 (4.4 to 5.2) | 401,075 (337,329 to 475,314) | 72.8 (62 to 85.2) | 218,153 (181,118 to 256,823) | 33.3 (28 to 38.7) | 19,408 (17,285 to 22,253) | 3.7 (3.3 to 4.2) | 495,039 (407,014 to 594,480) | 73.8 (61.8 to 87.6) |
|  | Female | 54,601 (45,654 to 63,915) | 33.6 (28.5 to 39.4) | 8,349 (7,297 to 9,382) | 7.1 (6.1 to 8) | 126,010 (105,267 to 149,152) | 74.2 (62.9 to 87.2) | 75,781 (63,789 to 88,228) | 27.9 (23.8 to 32.3) | 7,616 (6,835 to 8,772) | 3.7 (3.3 to 4.2) | 171,273 (143,215 to 203,599) | 61.2 (52.1 to 71.8) | 94,178 (78,196 to 111,357) | 27.9 (23.4 to 32.7) | 8,134 (6,986 to 9,618) | 2.9 (2.5 to 3.4) | 212,685 (174,599 to 256,032) | 61.9 (51.6 to 73.9) |
|  | Male | 82,448 (68,999 to 96,218) | 51.2 (43.9 to 59.9) | 11,910 (10,053 to 13,633) | 10.5 (8.7 to 12) | 196,777 (165,998 to 231,453) | 115.8 (98.5 to 135.7) | 100,881 (84,377 to 116,817) | 38.7 (33.2 to 44.5) | 11,230 (10,196 to 12,422) | 6.1 (5.5 to 6.6) | 229,802 (194,018 to 271,597) | 84.8 (72.5 to 99.1) | 123,975 (102,732 to 145,906) | 38.8 (32.6 to 45) | 11,274 (9,733 to 13,224) | 4.7 (4.1 to 5.5) | 282,354 (233,388 to 338,527) | 86 (71.9 to 102.1) |
| **GBD regions** | | | | | | | | | | | | | | | | | | | |
| East Asia | Both | 3,138 (2,624 to 3,606) | 68.6 (59.4 to 78.2) | 328 (251 to 404) | 11.5 (8.8 to 14.1) | 7,980 (6,760 to 9,322) | 161.3 (140.2 to 185.3) | 4,658 (3,912 to 5,392) | 58.7 (51 to 66.7) | 421 (343 to 512) | 8.4 (6.8 to 10.1) | 11,634 (9,913 to 13,675) | 136.2 (117.8 to 157.2) | 5,682 (4,785 to 6,571) | 56.6 (49 to 65.1) | 477 (383 to 601) | 7.2 (5.8 to 9) | 13,860 (11,737 to 16,267) | 129.9 (112.4 to 151.1) |
|  | Female | 854 (715 to 995) | 41.4 (35.3 to 47.6) | 111 (83 to 140) | 7.9 (5.7 to 9.9) | 2,068 (1,737 to 2,460) | 95.3 (81.2 to 110.8) | 1,403 (1,164 to 1,635) | 38 (32.4 to 43.6) | 158 (120 to 200) | 6.2 (4.5 to 8) | 3,418 (2,867 to 4,047) | 87.5 (75.6 to 102.2) | 1,826 (1,525 to 2,124) | 38.3 (32.8 to 44.3) | 181 (137 to 236) | 5.5 (4.1 to 7.1) | 4,366 (3,664 to 5,230) | 87.4 (74.3 to 102.7) |
|  | Male | 2,284 (1,917 to 2,647) | 94.6 (82.1 to 107.8) | 216 (160 to 280) | 15.3 (11.8 to 19.8) | 5,912 (4,972 to 6,999) | 223 (193.4 to 258.7) | 3,255 (2,715 to 3,823) | 78.2 (68.1 to 89.5) | 262 (208 to 329) | 10.7 (8.6 to 13) | 8,216 (6,933 to 9,761) | 181.4 (156.6 to 211.2) | 3,856 (3,234 to 4,505) | 73.9 (64.1 to 85.2) | 296 (232 to 378) | 9.1 (7.3 to 11.2) | 9,495 (8,005 to 11,271) | 169.3 (145.9 to 197.4) |
| Southeast Asia | Both | 29,106 (24,991 to 33,424) | 53 (45.7 to 61) | 1,960 (1,839 to 2,063) | 4.1 (3.8 to 4.3) | 69,745 (60,864 to 80,522) | 126.2 (109.3 to 144.9) | 31,598 (27,279 to 35,700) | 42.8 (37.6 to 47.8) | 2,518 (2,393 to 2,695) | 4.3 (4 to 4.6) | 74,449 (64,355 to 85,778) | 100.1 (87.5 to 114.2) | 35,398 (30,667 to 40,017) | 40.3 (35.1 to 45) | 2,445 (2,182 to 2,768) | 3.4 (3.1 to 3.9) | 82,145 (70,420 to 95,428) | 92.7 (79.8 to 106.9) |
|  | Female | 8,711 (7,461 to 9,995) | 29.8 (25.7 to 34.1) | 560 (516 to 600) | 2 (1.9 to 2.2) | 20,307 (17,341 to 23,575) | 69.2 (59.1 to 80) | 10,671 (9,151 to 12,288) | 28 (24.2 to 31.8) | 851 (797 to 946) | 2.7 (2.5 to 3) | 24,880 (21,214 to 29,146) | 64.8 (56.2 to 74.9) | 12,023 (10,233 to 13,800) | 26.4 (22.8 to 30.1) | 850 (742 to 983) | 2.3 (2 to 2.6) | 27,744 (23,464 to 32,634) | 60.4 (51.3 to 70.4) |
|  | Male | 20,395 (17,507 to 23,499) | 80.4 (69.4 to 92.9) | 1,400 (1,318 to 1,478) | 6.9 (6.5 to 7.3) | 49,438 (42,916 to 56,943) | 193.6 (169.1 to 221.3) | 20,927 (17,985 to 23,601) | 59.3 (51.8 to 66.3) | 1,666 (1,572 to 1,791) | 6.3 (6 to 6.9) | 49,570 (42,810 to 57,262) | 139.8 (123 to 159.3) | 23,375 (20,243 to 26,410) | 55.7 (49 to 62.4) | 1,596 (1,382 to 1,849) | 5 (4.3 to 5.7) | 54,402 (46,997 to 63,233) | 128.6 (111.8 to 148.1) |
| Oceania | Both | 69,495 (60,088 to 79,145) | 49.2 (42.6 to 55.8) | 7,174 (6,864 to 7,420) | 5.2 (5 to 5.4) | 161,728 (139,697 to 187,061) | 114 (98.1 to 132.1) | 75,457 (65,629 to 85,791) | 46.2 (39.9 to 52.2) | 6,127 (5,777 to 6,515) | 3.4 (3.2 to 3.6) | 178,018 (155,012 to 205,500) | 108.2 (93.8 to 124.6) | 75,532 (65,172 to 86,821) | 44.1 (37.9 to 50.4) | 5,923 (5,181 to 6,695) | 2.8 (2.4 to 3.1) | 174,814 (151,160 to 200,646) | 101.8 (87.9 to 118.3) |
|  | Female | 29,476 (25,092 to 34,228) | 38.4 (32.7 to 44.3) | 2,685 (2,526 to 2,832) | 3.4 (3.1 to 3.6) | 69,379 (59,176 to 81,011) | 90.1 (76.5 to 105.7) | 34,985 (30,025 to 40,220) | 38.8 (33.3 to 44.4) | 2,769 (2,525 to 3,002) | 2.4 (2.2 to 2.7) | 82,738 (71,467 to 95,425) | 91.5 (78.6 to 106.3) | 35,281 (29,961 to 40,910) | 37.7 (31.8 to 43.6) | 2,774 (2,348 to 3,186) | 2 (1.7 to 2.3) | 82,782 (71,585 to 95,983) | 88.6 (75.3 to 103.6) |
|  | Male | 40,019 (34,951 to 45,400) | 61.6 (54 to 69.5) | 4,488 (4,283 to 4,665) | 7.7 (7.3 to 8) | 92,349 (80,568 to 105,973) | 141.5 (123 to 162.5) | 40,473 (35,166 to 45,790) | 54.3 (47.6 to 61) | 3,359 (3,219 to 3,590) | 4.5 (4.3 to 4.9) | 95,280 (83,552 to 108,942) | 126.6 (110.6 to 145.4) | 40,251 (35,016 to 46,115) | 51.2 (44.4 to 58.2) | 3,149 (2,694 to 3,632) | 3.7 (3.1 to 4.2) | 92,031 (79,701 to 105,217) | 116.4 (101.3 to 133.7) |
| Central Asia | Both | 180,892 (148,494 to 217,046) | 69.7 (57.5 to 83) | 9,662 (9,131 to 10,102) | 3.6 (3.4 to 3.8) | 439,450 (359,261 to 532,691) | 168.1 (137.7 to 203.1) | 178,986 (148,538 to 211,442) | 65.5 (54.5 to 76.7) | 12,189 (11,799 to 12,476) | 4.1 (3.9 to 4.2) | 437,827 (365,173 to 523,256) | 158.7 (133 to 188.5) | 181,578 (150,357 to 219,896) | 65.2 (53.7 to 77.3) | 11,874 (10,503 to 13,274) | 3.6 (3.2 to 4) | 439,985 (363,829 to 533,969) | 157 (129.2 to 188.8) |
|  | Female | 65,938 (54,747 to 78,306) | 45.1 (37.1 to 53.5) | 2,990 (2,793 to 3,143) | 1.7 (1.6 to 1.8) | 160,939 (132,677 to 194,198) | 109.5 (89.9 to 132.2) | 72,109 (60,655 to 85,724) | 46.6 (38.7 to 54.7) | 4,421 (4,152 to 4,567) | 2.3 (2.1 to 2.3) | 176,441 (147,642 to 211,346) | 113.5 (94.5 to 135.2) | 77,824 (64,649 to 93,535) | 49.3 (40.6 to 58.5) | 4,925 (4,218 to 5,704) | 2.2 (1.9 to 2.6) | 188,197 (155,299 to 227,678) | 119.6 (98 to 143.7) |
|  | Male | 114,954 (93,902 to 138,794) | 101.4 (83.7 to 121.4) | 6,672 (6,197 to 7,053) | 6.7 (6.2 to 7) | 278,511 (225,577 to 336,745) | 244 (200.4 to 294.7) | 106,877 (88,463 to 126,615) | 88.9 (74.1 to 104.4) | 7,768 (7,545 to 7,985) | 6.7 (6.5 to 6.9) | 261,385 (218,206 to 312,806) | 214.8 (180.5 to 254.7) | 103,754 (84,878 to 125,834) | 84.6 (69.6 to 100.6) | 6,949 (5,941 to 8,105) | 5.5 (4.7 to 6.4) | 251,788 (206,536 to 305,753) | 202.9 (167.6 to 245.1) |
| Central Europe | Both | 102,382 (85,730 to 122,253) | 52.7 (44.3 to 62.2) | 7,224 (6,666 to 8,056) | 4.2 (3.8 to 4.7) | 245,335 (203,920 to 293,878) | 125.4 (104.6 to 149.4) | 127,393 (108,274 to 149,980) | 47.2 (39.8 to 55.1) | 5,235 (4,338 to 6,018) | 1.3 (1.1 to 1.5) | 309,239 (262,271 to 362,806) | 115.7 (97.3 to 137.4) | 136,724 (113,850 to 164,400) | 46.7 (38.5 to 55.5) | 5,642 (4,463 to 6,652) | 1 (0.8 to 1.1) | 335,708 (279,075 to 404,674) | 117.1 (95.4 to 141.6) |
|  | Female | 38,156 (31,736 to 45,799) | 35.8 (29.9 to 42.7) | 3,361 (2,988 to 3,708) | 3.3 (2.9 to 3.6) | 85,159 (71,199 to 101,857) | 80 (66.5 to 95.1) | 47,526 (40,351 to 55,939) | 29.4 (24.8 to 34.7) | 2,529 (1,890 to 3,061) | 0.9 (0.7 to 1) | 108,877 (92,893 to 128,616) | 68.5 (57.9 to 80.9) | 52,931 (44,047 to 63,735) | 29.3 (24.2 to 35.2) | 2,962 (2,123 to 3,704) | 0.7 (0.5 to 0.8) | 122,001 (101,359 to 147,218) | 69.5 (56.6 to 84.3) |
|  | Male | 64,226 (53,617 to 76,106) | 70.3 (59.1 to 83) | 3,862 (3,640 to 4,483) | 5.4 (5 to 6.2) | 160,176 (133,324 to 192,655) | 173.1 (144.8 to 206.3) | 79,866 (67,370 to 93,661) | 64.9 (54.3 to 75.7) | 2,706 (2,436 to 2,979) | 1.8 (1.6 to 2) | 200,362 (168,615 to 236,231) | 163 (136.8 to 192.7) | 83,793 (69,190 to 100,528) | 63.6 (51.9 to 75.7) | 2,680 (2,327 to 3,024) | 1.3 (1.1 to 1.4) | 213,707 (175,148 to 256,889) | 163.5 (133.1 to 196.9) |
| Eastern Europe | Both | 7,462 (6,275 to 8,610) | 32.5 (27.5 to 37.3) | 1,060 (950 to 1,144) | 4.8 (4.3 to 5.2) | 16,166 (13,904 to 18,692) | 70 (60.3 to 80.7) | 6,389 (5,437 to 7,434) | 18.3 (15.4 to 21.4) | 455 (384 to 519) | 1.1 (0.9 to 1.2) | 13,774 (11,546 to 16,232) | 40 (33.3 to 47.6) | 7,317 (6,154 to 8,705) | 17.8 (14.8 to 21.1) | 505 (414 to 594) | 0.9 (0.7 to 1) | 15,950 (13,143 to 19,216) | 39.6 (32 to 48) |
|  | Female | 3,630 (2,989 to 4,241) | 28.4 (23.5 to 33) | 552 (473 to 606) | 4 (3.5 to 4.4) | 7,788 (6,662 to 9,108) | 61 (51.9 to 71.6) | 3,063 (2,575 to 3,611) | 16.2 (13.4 to 19.2) | 240 (190 to 281) | 0.9 (0.7 to 1) | 6,538 (5,434 to 7,753) | 35.3 (28.9 to 42.4) | 3,536 (2,958 to 4,238) | 16 (13.2 to 19.2) | 260 (198 to 314) | 0.8 (0.6 to 0.9) | 7,619 (6,245 to 9,287) | 35.4 (28.3 to 43.2) |
|  | Male | 3,831 (3,222 to 4,448) | 37 (31.4 to 43.1) | 509 (464 to 552) | 5.9 (5.3 to 6.3) | 8,377 (7,177 to 9,708) | 79.9 (68.5 to 92.2) | 3,325 (2,817 to 3,856) | 20.6 (17.4 to 24) | 215 (188 to 245) | 1.3 (1.1 to 1.4) | 7,236 (6,024 to 8,631) | 44.8 (37.1 to 53.7) | 3,781 (3,173 to 4,451) | 19.7 (16.3 to 23.5) | 245 (204 to 291) | 1 (0.9 to 1.2) | 8,331 (6,840 to 10,026) | 44 (35.4 to 53.4) |
| High-income Asia Pacific | Both | 141,599 (122,410 to 163,120) | 26.8 (23.2 to 30.9) | 23,818 (21,802 to 24,947) | 4.1 (3.7 to 4.3) | 299,185 (257,782 to 346,968) | 57.3 (48.8 to 66.8) | 129,868 (112,086 to 148,918) | 20 (17.1 to 22.9) | 13,495 (11,978 to 14,917) | 1.5 (1.4 to 1.7) | 282,155 (242,000 to 326,600) | 44.4 (37.7 to 52) | 124,178 (104,988 to 146,343) | 17.9 (14.9 to 21.2) | 13,345 (11,615 to 14,955) | 1.2 (1.1 to 1.4) | 267,120 (222,158 to 318,412) | 40 (32.5 to 48.3) |
|  | Female | 70,286 (59,869 to 81,531) | 23 (19.5 to 26.6) | 11,963 (10,547 to 12,686) | 3.1 (2.8 to 3.3) | 148,442 (127,441 to 173,138) | 49.7 (42 to 58.6) | 63,835 (54,676 to 73,689) | 17.6 (14.9 to 20.4) | 7,148 (6,122 to 8,105) | 1.3 (1.1 to 1.4) | 137,942 (118,405 to 161,464) | 39.5 (33.2 to 46.8) | 60,852 (51,205 to 72,419) | 16.1 (13.2 to 19.2) | 6,981 (5,803 to 7,947) | 1 (0.9 to 1.1) | 130,323 (107,168 to 156,759) | 36 (28.7 to 43.8) |
|  | Male | 71,313 (62,083 to 82,001) | 31.5 (27.5 to 36.2) | 11,855 (11,038 to 12,324) | 5.5 (5.1 to 5.8) | 150,742 (129,255 to 175,243) | 66 (56.5 to 76.6) | 66,033 (57,429 to 75,246) | 22.5 (19.4 to 25.6) | 6,347 (5,871 to 6,948) | 1.9 (1.8 to 2.1) | 144,213 (123,721 to 166,347) | 49.7 (42.4 to 58.2) | 63,326 (53,673 to 74,083) | 19.9 (16.6 to 23.4) | 6,363 (5,701 to 7,122) | 1.5 (1.4 to 1.7) | 136,797 (114,251 to 161,861) | 44.1 (36.1 to 53.1) |
| Australasia | Both | 9,104 (7,840 to 10,357) | 19.5 (16.8 to 22.1) | 1,412 (1,296 to 1,525) | 3.3 (3 to 3.5) | 19,860 (16,937 to 23,199) | 42.1 (35.9 to 49) | 11,082 (9,555 to 12,569) | 16.7 (14.3 to 19) | 1,166 (1,049 to 1,279) | 1.7 (1.5 to 1.8) | 24,077 (20,372 to 28,099) | 36.4 (30.7 to 42.5) | 12,596 (10,682 to 14,493) | 16.2 (13.7 to 18.7) | 1,312 (1,141 to 1,499) | 1.5 (1.3 to 1.8) | 27,349 (22,907 to 32,304) | 35.4 (29.5 to 42) |
|  | Female | 3,718 (3,115 to 4,294) | 14.8 (12.4 to 17.1) | 517 (466 to 563) | 2.1 (1.9 to 2.3) | 8,133 (6,786 to 9,650) | 32.3 (26.9 to 38.4) | 4,721 (3,979 to 5,442) | 13 (11 to 15.1) | 493 (428 to 551) | 1.2 (1 to 1.3) | 10,252 (8,590 to 12,019) | 28.7 (23.8 to 33.9) | 5,508 (4,618 to 6,445) | 13.1 (10.8 to 15.4) | 578 (490 to 669) | 1.1 (1 to 1.3) | 11,958 (9,869 to 14,307) | 28.8 (23.3 to 34.7) |
|  | Male | 5,386 (4,664 to 6,137) | 24.9 (21.6 to 28.2) | 895 (820 to 976) | 4.8 (4.4 to 5.2) | 11,727 (10,045 to 13,662) | 53.3 (45.7 to 61.7) | 6,360 (5,510 to 7,255) | 20.8 (18.1 to 23.7) | 673 (605 to 749) | 2.3 (2.1 to 2.6) | 13,825 (11,742 to 16,036) | 45 (38.3 to 52.1) | 7,088 (6,015 to 8,123) | 19.8 (16.8 to 22.6) | 735 (630 to 867) | 2.1 (1.8 to 2.5) | 15,391 (12,939 to 18,170) | 42.8 (36.1 to 50.8) |
| Western Europe | Both | 186,171 (153,781 to 224,413) | 54.9 (45.6 to 66.2) | 9,721 (8,902 to 10,203) | 2.7 (2.4 to 2.8) | 436,149 (363,549 to 524,616) | 128.5 (107 to 154.1) | 196,723 (171,605 to 225,326) | 42.8 (37.4 to 48.6) | 5,018 (4,462 to 5,418) | 0.9 (0.8 to 1) | 462,168 (406,387 to 528,812) | 100.1 (88.2 to 114.2) | 229,019 (195,794 to 270,045) | 42.5 (36.2 to 49.8) | 5,698 (5,040 to 6,154) | 0.9 (0.8 to 0.9) | 541,480 (466,230 to 632,345) | 100.6 (86.7 to 116.5) |
|  | Female | 101,280 (83,497 to 122,306) | 53.3 (43.9 to 64.4) | 5,025 (4,390 to 5,367) | 2.2 (1.9 to 2.3) | 238,298 (198,419 to 285,940) | 125.1 (103.7 to 150.1) | 106,383 (92,409 to 122,021) | 43.3 (37.6 to 49.3) | 2,604 (2,199 to 2,889) | 0.8 (0.7 to 0.8) | 256,099 (225,113 to 292,764) | 103.7 (91.4 to 117.8) | 123,468 (105,422 to 146,208) | 43.9 (37.3 to 51.3) | 2,879 (2,440 to 3,193) | 0.7 (0.6 to 0.8) | 297,343 (256,196 to 347,786) | 105.5 (90.7 to 122.6) |
|  | Male | 84,891 (70,261 to 102,354) | 57 (47.5 to 68.3) | 4,696 (4,381 to 4,913) | 3.4 (3.1 to 3.5) | 197,852 (164,529 to 237,781) | 132.9 (111.3 to 159.3) | 90,339 (78,954 to 103,257) | 42.6 (37.5 to 48.5) | 2,414 (2,212 to 2,583) | 1.1 (1 to 1.2) | 206,069 (180,805 to 236,237) | 97.2 (85.5 to 111.1) | 105,552 (89,906 to 124,653) | 41.5 (35.5 to 48.6) | 2,819 (2,559 to 3,036) | 1 (0.9 to 1.1) | 244,137 (209,457 to 285,289) | 96.3 (83.4 to 111.2) |
| Southern Latin America | Both | 9,767 (8,511 to 11,023) | 34 (29.8 to 38.2) | 1,732 (1,537 to 1,967) | 6.7 (6 to 7.5) | 21,482 (18,559 to 24,850) | 73.1 (63.4 to 83.9) | 11,498 (9,986 to 13,018) | 26.3 (22.9 to 29.7) | 1,595 (1,362 to 1,872) | 3.8 (3.2 to 4.4) | 24,813 (21,358 to 28,912) | 56.4 (48.7 to 65.5) | 13,242 (11,452 to 15,113) | 26.2 (22.6 to 29.9) | 1,759 (1,476 to 2,092) | 3.4 (2.9 to 4.1) | 28,403 (24,106 to 33,183) | 56.3 (47.7 to 66.2) |
|  | Female | 3,617 (3,127 to 4,128) | 24.5 (21.3 to 27.7) | 613 (491 to 761) | 4.6 (3.8 to 5.5) | 8,101 (6,927 to 9,451) | 53.7 (46 to 62.3) | 4,672 (3,997 to 5,359) | 20.6 (17.6 to 23.6) | 620 (488 to 770) | 2.7 (2.1 to 3.4) | 10,332 (8,767 to 12,154) | 45.5 (38.6 to 53.4) | 5,399 (4,626 to 6,194) | 20.5 (17.5 to 23.5) | 680 (539 to 855) | 2.5 (1.9 to 3.1) | 11,848 (9,962 to 14,003) | 45.3 (38 to 53.5) |
|  | Male | 6,150 (5,384 to 6,933) | 44.1 (38.8 to 49.7) | 1,119 (956 to 1,281) | 9 (7.8 to 10.3) | 13,381 (11,614 to 15,371) | 93.7 (81.5 to 106.9) | 6,826 (5,974 to 7,689) | 32.4 (28.5 to 36.4) | 976 (805 to 1,180) | 5 (4.1 to 6) | 14,481 (12,505 to 16,741) | 68 (58.8 to 78.4) | 7,842 (6,795 to 8,961) | 32.4 (28 to 36.9) | 1,078 (881 to 1,285) | 4.5 (3.7 to 5.4) | 16,555 (14,100 to 19,263) | 68.1 (58.2 to 79.1) |
| High-income North America | Both | 8,048 (6,942 to 9,134) | 32.6 (28.6 to 36.8) | 1,659 (1,427 to 1,891) | 8.2 (7 to 9.3) | 17,462 (15,083 to 20,397) | 67.2 (58.4 to 76.8) | 10,426 (9,154 to 11,660) | 23 (20.4 to 25.5) | 1,636 (1,410 to 1,918) | 4.1 (3.5 to 4.8) | 21,862 (18,972 to 25,184) | 47.2 (41.3 to 53.8) | 12,367 (10,675 to 14,070) | 21 (18.2 to 23.7) | 1,620 (1,297 to 2,004) | 3 (2.4 to 3.7) | 25,932 (22,164 to 30,509) | 43.4 (37.3 to 50.6) |
|  | Female | 3,513 (3,020 to 4,007) | 28.4 (24.7 to 32.3) | 686 (576 to 803) | 6.7 (5.7 to 7.8) | 7,677 (6,552 to 9,054) | 58.8 (50.7 to 67.7) | 4,905 (4,276 to 5,511) | 21.3 (18.8 to 23.9) | 752 (629 to 933) | 3.7 (3.1 to 4.5) | 10,337 (8,981 to 11,887) | 44.2 (38.4 to 50.5) | 5,963 (5,176 to 6,787) | 19.8 (17.2 to 22.5) | 785 (616 to 998) | 2.8 (2.2 to 3.5) | 12,492 (10,700 to 14,583) | 41.1 (35.5 to 47.7) |
|  | Male | 4,535 (3,911 to 5,137) | 36.9 (32.4 to 41.9) | 974 (791 to 1,150) | 9.8 (7.8 to 11.4) | 9,785 (8,474 to 11,459) | 75.8 (66.1 to 87.2) | 5,521 (4,827 to 6,189) | 24.6 (21.8 to 27.4) | 884 (758 to 1,032) | 4.6 (4 to 5.4) | 11,525 (9,980 to 13,328) | 50.2 (43.9 to 57.3) | 6,404 (5,482 to 7,376) | 22.1 (19.1 to 25.2) | 835 (649 to 1,061) | 3.3 (2.5 to 4.1) | 13,440 (11,433 to 16,024) | 45.5 (38.9 to 53.5) |
| Caribbean | Both | 26,916 (23,235 to 30,772) | 28.9 (25.2 to 33.5) | 7,143 (6,721 to 7,416) | 9.4 (8.7 to 9.8) | 53,255 (45,666 to 62,250) | 53.1 (45.8 to 61.4) | 37,045 (32,695 to 41,716) | 19.9 (17.7 to 22.4) | 6,688 (6,103 to 7,166) | 4.1 (3.7 to 4.4) | 77,000 (66,576 to 88,212) | 40 (34.9 to 45.7) | 40,821 (35,235 to 47,183) | 17 (14.7 to 19.6) | 7,857 (6,819 to 9,139) | 3.5 (3 to 4.1) | 80,456 (68,954 to 94,070) | 33 (28.5 to 38.5) |
|  | Female | 11,966 (10,325 to 13,829) | 25.9 (22.4 to 30.2) | 3,262 (3,015 to 3,421) | 8.5 (7.7 to 9) | 23,864 (20,423 to 27,738) | 47.9 (41.2 to 55.8) | 17,360 (15,271 to 19,683) | 17.8 (15.7 to 20.2) | 3,188 (2,855 to 3,505) | 3.7 (3.3 to 4) | 36,871 (31,857 to 42,282) | 36.7 (31.9 to 41.9) | 19,446 (16,731 to 22,581) | 15.2 (13.1 to 17.7) | 3,858 (3,281 to 4,523) | 3.1 (2.7 to 3.6) | 38,333 (32,633 to 44,809) | 29.7 (25.4 to 34.6) |
|  | Male | 14,949 (12,897 to 17,045) | 32.1 (28 to 36.8) | 3,881 (3,638 to 4,041) | 10.3 (9.5 to 10.8) | 29,390 (25,022 to 34,577) | 58.4 (50.5 to 68.1) | 19,685 (17,377 to 22,288) | 22.2 (19.6 to 25) | 3,500 (3,219 to 3,769) | 4.6 (4.2 to 5) | 40,128 (34,885 to 46,087) | 43.6 (38.2 to 49.8) | 21,375 (18,450 to 24,648) | 19 (16.4 to 21.8) | 3,999 (3,372 to 4,742) | 3.9 (3.3 to 4.6) | 42,123 (36,072 to 49,209) | 36.6 (31.5 to 42.7) |
| Andean Latin America | Both | 72,775 (60,374 to 85,984) | 67 (56 to 80.3) | 5,073 (4,769 to 5,326) | 6 (5.6 to 6.4) | 171,526 (142,478 to 204,817) | 157.7 (131.9 to 189.6) | 57,064 (48,528 to 66,626) | 29.5 (25.1 to 34.5) | 4,734 (4,386 to 5,103) | 2.7 (2.5 to 3) | 128,587 (109,347 to 150,775) | 66 (56.5 to 77.2) | 52,581 (43,927 to 63,390) | 21.6 (18.1 to 26) | 5,286 (4,772 to 5,823) | 2.3 (2 to 2.5) | 115,939 (96,300 to 140,459) | 47.3 (39.6 to 57.1) |
|  | Female | 29,580 (24,614 to 34,910) | 52.1 (43.5 to 62.3) | 1,760 (1,598 to 1,857) | 4.2 (3.7 to 4.4) | 69,770 (57,876 to 83,913) | 122.5 (101.7 to 147.1) | 24,367 (20,726 to 28,547) | 23.8 (20.3 to 28) | 1,856 (1,656 to 2,065) | 2 (1.7 to 2.2) | 54,470 (46,313 to 63,669) | 52.9 (45.1 to 61.9) | 22,864 (19,004 to 27,554) | 17.6 (14.6 to 21) | 2,153 (1,875 to 2,439) | 1.6 (1.4 to 1.8) | 50,900 (42,412 to 61,547) | 39.1 (32.7 to 47.2) |
|  | Male | 43,196 (35,852 to 51,245) | 83.5 (69.4 to 100.6) | 3,314 (3,113 to 3,501) | 8.2 (7.6 to 8.6) | 101,756 (84,518 to 121,563) | 196.8 (164.7 to 236) | 32,697 (27,696 to 37,943) | 35.8 (30.5 to 41.9) | 2,878 (2,710 to 3,115) | 3.7 (3.4 to 4) | 74,117 (63,195 to 87,163) | 80.9 (69.4 to 94.6) | 29,717 (24,867 to 35,759) | 26.3 (22.1 to 31.5) | 3,132 (2,854 to 3,469) | 3 (2.7 to 3.4) | 65,039 (54,076 to 78,454) | 56.7 (47.5 to 68.5) |
| Central Latin America | Both | 106,887 (88,692 to 124,469) | 44.9 (38.1 to 51.8) | 9,069 (7,606 to 11,383) | 5.8 (4.7 to 7.6) | 255,741 (213,148 to 308,510) | 104 (88.2 to 122.2) | 179,187 (146,892 to 211,197) | 40.3 (33.9 to 46.6) | 9,191 (7,870 to 10,848) | 3.3 (2.8 to 3.9) | 446,676 (369,698 to 541,181) | 97 (82.4 to 115.3) | 215,511 (175,086 to 256,243) | 38.6 (32.1 to 45) | 9,604 (7,925 to 11,651) | 2.6 (2.1 to 3.1) | 522,218 (424,465 to 637,549) | 91.1 (75.3 to 109.9) |
|  | Female | 106,887 (88,692 to 124,469) | 44.9 (38.1 to 51.8) | 9,069 (7,606 to 11,383) | 5.8 (4.7 to 7.6) | 255,741 (213,148 to 308,510) | 104 (88.2 to 122.2) | 179,187 (146,892 to 211,197) | 40.3 (33.9 to 46.6) | 9,191 (7,870 to 10,848) | 3.3 (2.8 to 3.9) | 446,676 (369,698 to 541,181) | 97 (82.4 to 115.3) | 215,511 (175,086 to 256,243) | 38.6 (32.1 to 45) | 9,604 (7,925 to 11,651) | 2.6 (2.1 to 3.1) | 522,218 (424,465 to 637,549) | 91.1 (75.3 to 109.9) |
|  | Male | 49,999 (41,032 to 58,811) | 42.2 (35.4 to 48.8) | 3,765 (2,703 to 5,012) | 4.9 (3.4 to 7) | 121,908 (100,503 to 148,604) | 100 (84.3 to 119) | 88,710 (72,218 to 104,645) | 41.1 (34.4 to 47.8) | 4,032 (3,282 to 5,041) | 3 (2.4 to 3.8) | 227,437 (186,871 to 278,408) | 102.3 (86.4 to 121.9) | 105,446 (85,040 to 125,867) | 39.4 (32.4 to 46.4) | 4,237 (3,471 to 5,227) | 2.4 (1.9 to 3) | 260,143 (209,528 to 316,891) | 95 (78.1 to 115) |
| Tropical Latin America | Both | 49,999 (41,032 to 58,811) | 42.2 (35.4 to 48.8) | 3,765 (2,703 to 5,012) | 4.9 (3.4 to 7) | 121,908 (100,503 to 148,604) | 100 (84.3 to 119) | 88,710 (72,218 to 104,645) | 41.1 (34.4 to 47.8) | 4,032 (3,282 to 5,041) | 3 (2.4 to 3.8) | 227,437 (186,871 to 278,408) | 102.3 (86.4 to 121.9) | 105,446 (85,040 to 125,867) | 39.4 (32.4 to 46.4) | 4,237 (3,471 to 5,227) | 2.4 (1.9 to 3) | 260,143 (209,528 to 316,891) | 95 (78.1 to 115) |
|  | Female | 56,888 (47,640 to 66,088) | 47.6 (40.8 to 54.9) | 5,303 (4,225 to 6,666) | 6.7 (5.1 to 8.8) | 133,833 (112,608 to 159,476) | 107.7 (91.8 to 126.2) | 90,478 (74,105 to 106,514) | 39.5 (33.5 to 45.5) | 5,158 (4,308 to 6,092) | 3.6 (2.9 to 4.2) | 219,239 (182,463 to 265,505) | 91.9 (78.4 to 109) | 110,065 (89,543 to 130,199) | 37.8 (31.7 to 43.8) | 5,368 (4,260 to 6,534) | 2.8 (2.2 to 3.4) | 262,075 (214,188 to 320,663) | 87.3 (72.4 to 105.4) |
|  | Male | 56,888 (47,640 to 66,088) | 47.6 (40.8 to 54.9) | 5,303 (4,225 to 6,666) | 6.7 (5.1 to 8.8) | 133,833 (112,608 to 159,476) | 107.7 (91.8 to 126.2) | 90,478 (74,105 to 106,514) | 39.5 (33.5 to 45.5) | 5,158 (4,308 to 6,092) | 3.6 (2.9 to 4.2) | 219,239 (182,463 to 265,505) | 91.9 (78.4 to 109) | 110,065 (89,543 to 130,199) | 37.8 (31.7 to 43.8) | 5,368 (4,260 to 6,534) | 2.8 (2.2 to 3.4) | 262,075 (214,188 to 320,663) | 87.3 (72.4 to 105.4) |
| North Africa and Middle East | Both | 928,339 (778,472 to 1,097,471) | 135.9 (115.2 to 161.6) | 95,677 (82,758 to 108,458) | 17.9 (15.4 to 20.2) | 2,067,961 (1,737,831 to 2,452,046) | 277.7 (232.5 to 329.8) | 1,001,352 (840,706 to 1,167,960) | 84.4 (72 to 98.7) | 75,071 (68,118 to 83,753) | 8 (7.2 to 9) | 2,133,781 (1,805,376 to 2,520,967) | 168.5 (142.6 to 197.4) | 1,193,531 (999,122 to 1,404,393) | 77.5 (65.3 to 91.6) | 74,929 (63,470 to 89,532) | 5.9 (5 to 7.1) | 2,520,121 (2,085,276 to 3,013,098) | 156.6 (130.6 to 187) |
|  | Female | 928,339 (778,472 to 1,097,471) | 135.9 (115.2 to 161.6) | 95,677 (82,758 to 108,458) | 17.9 (15.4 to 20.2) | 2,067,961 (1,737,831 to 2,452,046) | 277.7 (232.5 to 329.8) | 1,001,352 (840,706 to 1,167,960) | 84.4 (72 to 98.7) | 75,071 (68,118 to 83,753) | 8 (7.2 to 9) | 2,133,781 (1,805,376 to 2,520,967) | 168.5 (142.6 to 197.4) | 1,193,531 (999,122 to 1,404,393) | 77.5 (65.3 to 91.6) | 74,929 (63,470 to 89,532) | 5.9 (5 to 7.1) | 2,520,121 (2,085,276 to 3,013,098) | 156.6 (130.6 to 187) |
|  | Male | 433,157 (362,878 to 509,677) | 132.1 (112.5 to 156.7) | 39,318 (30,907 to 48,324) | 16.4 (12.9 to 20.1) | 991,561 (829,549 to 1,169,171) | 270.7 (228 to 318.4) | 519,895 (440,459 to 606,254) | 88.1 (75.4 to 102.7) | 36,447 (30,844 to 42,080) | 8 (6.7 to 9.3) | 1,132,799 (950,938 to 1,342,207) | 178.7 (151.3 to 209.3) | 643,325 (538,660 to 754,966) | 83.3 (70.2 to 98.2) | 39,032 (31,039 to 48,043) | 6.1 (4.8 to 7.5) | 1,382,313 (1,147,072 to 1,651,068) | 171.4 (143.5 to 203.2) |
| South Asia | Both | 433,157 (362,878 to 509,677) | 132.1 (112.5 to 156.7) | 39,318 (30,907 to 48,324) | 16.4 (12.9 to 20.1) | 991,561 (829,549 to 1,169,171) | 270.7 (228 to 318.4) | 519,895 (440,459 to 606,254) | 88.1 (75.4 to 102.7) | 36,447 (30,844 to 42,080) | 8 (6.7 to 9.3) | 1,132,799 (950,938 to 1,342,207) | 178.7 (151.3 to 209.3) | 643,325 (538,660 to 754,966) | 83.3 (70.2 to 98.2) | 39,032 (31,039 to 48,043) | 6.1 (4.8 to 7.5) | 1,382,313 (1,147,072 to 1,651,068) | 171.4 (143.5 to 203.2) |
|  | Female | 495,182 (413,057 to 591,631) | 138.9 (117.1 to 167.2) | 56,358 (43,926 to 64,677) | 19.2 (14.8 to 22) | 1,076,400 (888,768 to 1,287,560) | 282.8 (235 to 341.4) | 481,457 (404,455 to 563,527) | 80.5 (68.6 to 94.6) | 38,624 (34,542 to 44,313) | 8 (7.1 to 9) | 1,000,982 (838,193 to 1,182,737) | 158 (133.2 to 186.8) | 550,207 (458,797 to 652,603) | 71.3 (60.1 to 84.5) | 35,897 (28,289 to 45,139) | 5.7 (4.5 to 7.1) | 1,137,808 (938,608 to 1,366,303) | 141.5 (116.6 to 169.5) |
|  | Male | 495,182 (413,057 to 591,631) | 138.9 (117.1 to 167.2) | 56,358 (43,926 to 64,677) | 19.2 (14.8 to 22) | 1,076,400 (888,768 to 1,287,560) | 282.8 (235 to 341.4) | 481,457 (404,455 to 563,527) | 80.5 (68.6 to 94.6) | 38,624 (34,542 to 44,313) | 8 (7.1 to 9) | 1,000,982 (838,193 to 1,182,737) | 158 (133.2 to 186.8) | 550,207 (458,797 to 652,603) | 71.3 (60.1 to 84.5) | 35,897 (28,289 to 45,139) | 5.7 (4.5 to 7.1) | 1,137,808 (938,608 to 1,366,303) | 141.5 (116.6 to 169.5) |
| Central Sub-Saharan Africa | Both | 18,307 (15,491 to 20,964) | 58.3 (50.8 to 66.2) | 1,963 (1,491 to 2,480) | 8.4 (5.4 to 11.3) | 40,708 (34,502 to 47,693) | 118.7 (102.4 to 137.1) | 32,978 (27,937 to 38,049) | 57.6 (50.8 to 65.1) | 2,888 (2,152 to 3,729) | 7.3 (4.6 to 10) | 73,530 (62,911 to 86,003) | 117.7 (101.7 to 135) | 43,586 (36,675 to 50,392) | 56.5 (49.5 to 64.3) | 2,998 (2,139 to 3,997) | 5.8 (3.6 to 8.3) | 97,138 (81,817 to 115,774) | 116.5 (99.9 to 135.4) |
|  | Female | 9,274 (7,745 to 10,674) | 57.1 (49.6 to 65) | 773 (484 to 1,079) | 7.5 (3.5 to 11.2) | 21,173 (17,882 to 24,950) | 118.9 (102.4 to 137.6) | 17,767 (14,991 to 20,488) | 58.4 (51.2 to 66.2) | 1,466 (935 to 2,027) | 7.4 (3.6 to 11.1) | 40,293 (34,332 to 47,246) | 122.3 (106 to 140.1) | 23,246 (19,534 to 26,969) | 57.3 (49.9 to 65.3) | 1,517 (953 to 2,139) | 6 (3.1 to 9.1) | 52,806 (44,216 to 63,379) | 121.1 (104.2 to 141.1) |
|  | Male | 9,034 (7,700 to 10,364) | 59.8 (51.8 to 68.2) | 1,190 (840 to 1,557) | 9.4 (6.3 to 12.4) | 19,535 (16,545 to 22,852) | 118.9 (101.2 to 138.3) | 15,211 (12,917 to 17,482) | 55.8 (49.1 to 63.2) | 1,422 (925 to 2,021) | 6.4 (4.3 to 9.2) | 33,237 (28,147 to 38,947) | 111.2 (96.3 to 128.2) | 20,339 (17,172 to 23,490) | 54.7 (47.6 to 62.6) | 1,481 (929 to 2,185) | 5 (3.1 to 7.2) | 44,331 (37,222 to 52,663) | 109.7 (93.6 to 128.4) |
| Eastern Sub-Saharan Africa | Both | 66,719 (54,999 to 78,848) | 55.7 (47.2 to 64.3) | 6,112 (4,515 to 8,535) | 7.3 (5.3 to 10.2) | 155,761 (129,352 to 186,016) | 124.8 (106.5 to 145.6) | 96,290 (79,559 to 113,836) | 46.2 (39.5 to 52.7) | 6,581 (4,826 to 8,866) | 4.9 (3.3 to 6.8) | 223,851 (186,458 to 266,402) | 102.8 (88.6 to 119.2) | 128,416 (104,537 to 151,986) | 46.4 (39.4 to 53.3) | 7,324 (4,936 to 9,942) | 4.1 (2.6 to 5.9) | 298,069 (247,371 to 359,431) | 103.9 (88.9 to 121.3) |
|  | Female | 30,195 (24,542 to 35,844) | 50.6 (43.1 to 58.3) | 1,985 (1,388 to 2,963) | 5.1 (3.5 to 7.7) | 70,924 (58,578 to 85,835) | 112.7 (95.7 to 131.1) | 42,571 (34,953 to 50,372) | 41.7 (35.8 to 47.7) | 2,117 (1,421 to 3,050) | 3.4 (2.1 to 5.1) | 98,086 (80,779 to 118,724) | 91.1 (77.8 to 105.2) | 57,427 (46,766 to 68,251) | 42.4 (36 to 49.1) | 2,428 (1,558 to 3,517) | 3 (1.8 to 4.5) | 132,364 (108,229 to 161,152) | 93.5 (79.1 to 109.3) |
|  | Male | 36,524 (30,121 to 42,808) | 61 (51.7 to 70.4) | 4,127 (2,895 to 5,931) | 9.6 (6.9 to 13.4) | 84,838 (70,752 to 100,578) | 137.1 (116.3 to 161.1) | 53,719 (44,632 to 63,065) | 50.5 (43.1 to 57.7) | 4,464 (3,272 to 6,097) | 6.5 (4.4 to 8.8) | 125,765 (105,678 to 148,806) | 114.1 (98.7 to 132.6) | 70,989 (57,956 to 84,061) | 50.1 (42.2 to 57.7) | 4,896 (3,294 to 6,766) | 5.3 (3.3 to 7.6) | 165,705 (138,196 to 199,348) | 113.7 (97.5 to 133.5) |
| Southern Sub-Saharan Africa | Both | 14,646 (12,123 to 17,114) | 39 (32.9 to 45.3) | 1,487 (1,063 to 1,911) | 5.5 (3.9 to 7.2) | 32,910 (27,126 to 39,912) | 84.6 (71.1 to 100) | 22,384 (18,769 to 25,912) | 39 (33.4 to 44.8) | 2,754 (2,480 to 3,105) | 6.7 (6.1 to 7.4) | 50,756 (42,454 to 60,265) | 84.9 (72 to 99.4) | 25,658 (21,394 to 29,846) | 38.1 (32.2 to 44.3) | 2,513 (2,241 to 2,852) | 4.9 (4.4 to 5.5) | 57,827 (47,764 to 69,641) | 82.9 (69.2 to 98) |
|  | Female | 6,765 (5,530 to 7,967) | 32.9 (27.7 to 38.2) | 604 (427 to 769) | 4.1 (2.8 to 5.2) | 15,268 (12,374 to 18,775) | 71.7 (59.4 to 85.7) | 11,304 (9,456 to 13,145) | 36.1 (30.7 to 41.8) | 1,283 (1,145 to 1,461) | 5.4 (4.8 to 6.1) | 25,639 (21,199 to 30,992) | 79.2 (66.6 to 93.1) | 12,854 (10,743 to 15,047) | 35.5 (29.9 to 41.5) | 1,154 (1,012 to 1,336) | 4 (3.5 to 4.6) | 28,829 (23,535 to 34,805) | 77.4 (63.9 to 92.9) |
|  | Male | 7,881 (6,535 to 9,211) | 45.8 (39 to 53.4) | 883 (610 to 1,196) | 7.3 (5.1 to 10) | 17,642 (14,702 to 21,132) | 99 (83.7 to 116.2) | 11,080 (9,310 to 12,800) | 42.1 (36.1 to 48.6) | 1,471 (1,302 to 1,718) | 8.1 (7.3 to 9.5) | 25,118 (21,275 to 29,647) | 91.1 (77.8 to 106.5) | 12,804 (10,573 to 14,959) | 40.6 (34.1 to 47.1) | 1,359 (1,115 to 1,624) | 6 (5.1 to 7) | 28,998 (24,119 to 34,732) | 88.3 (74.2 to 104.7) |
| Western Sub-Saharan Africa | Both | 85,116 (70,575 to 99,889) | 62.9 (54 to 72.4) | 8,338 (6,373 to 11,430) | 9.4 (7.2 to 12.8) | 204,168 (170,637 to 243,160) | 140.8 (120.5 to 164.1) | 149,419 (123,308 to 174,962) | 59.5 (51.3 to 68) | 11,671 (8,508 to 17,011) | 8 (5.9 to 11.4) | 364,599 (303,428 to 432,984) | 135.3 (116.4 to 156.7) | 189,288 (154,410 to 223,298) | 58.2 (49.9 to 66.9) | 12,672 (9,247 to 16,901) | 6.7 (5.1 to 8.6) | 461,063 (379,646 to 552,951) | 132.3 (113 to 154.3) |
|  | Female | 48,294 (39,456 to 57,769) | 67.8 (57.7 to 78.4) | 3,593 (2,648 to 4,769) | 8 (5.8 to 10.6) | 118,142 (95,536 to 143,757) | 154 (131.1 to 180.7) | 92,630 (75,600 to 110,327) | 70.5 (60.5 to 81.3) | 5,348 (3,866 to 7,624) | 7.5 (5.5 to 10.6) | 231,421 (188,292 to 280,453) | 163.2 (139.6 to 190.3) | 117,987 (95,368 to 141,258) | 68.5 (58.1 to 79.3) | 6,112 (4,489 to 8,434) | 6.5 (4.9 to 8.7) | 295,010 (238,937 to 359,123) | 158.5 (134.8 to 186.3) |
|  | Male | 36,822 (30,694 to 42,904) | 56.4 (48.3 to 65) | 4,744 (2,978 to 7,320) | 10.5 (6.7 to 16.2) | 86,026 (72,478 to 101,874) | 124.3 (106.4 to 145.5) | 56,788 (47,348 to 66,277) | 48.1 (41.6 to 54.7) | 6,323 (4,138 to 9,647) | 8.4 (5.6 to 12.4) | 133,178 (112,806 to 157,395) | 106.7 (92.7 to 123.1) | 71,302 (58,665 to 83,958) | 47.2 (40.4 to 54.1) | 6,560 (4,525 to 9,104) | 6.9 (4.9 to 9.3) | 166,053 (138,809 to 198,682) | 104.5 (89.6 to 122.2) |

Data in parenthesis are 95% uncertainty intervals (95% UI)
