# Supplementary material for: Quality of care of peptic ulcer disease worldwide: A systematic analysis for the global burden of disease study 1990–2019
Source: PLoS One. 2022 Aug 1;17(8):e0271284. doi: 10.1371/journal.pone.0271284 (PMC9342757; doi:10.1371/journal.pone.0271284)
Supplement: S4 Table — (DOCX) [file pone.0271284.s004.docx]

| Location | Sex | **1990 to 2010** | | | | | | **2010 to 2019** | | | | | | **1990 to 2019** | | | | | |
| --- | --- | --- | --- | --- | --- | --- | --- | --- | --- | --- | --- | --- | --- | --- | --- | --- | --- | --- | --- |
|  |  | **Incidence** | | **Deaths** | | **Prevalence** | | **Incidence** | | **Deaths** | | **Prevalence** | | **Incidence** | | **Deaths** | | **Prevalence** | |
|  |  | **Number** | **Rate** | **Number** | **Rate** | **Number** | **Rate** | **Number** | **Rate** | **Number** | **Rate** | **Number** | **Rate** | **Number** | **Rate** | **Number** | **Rate** | **Number** | **Rate** |
| Global | Both | 10.4 (7.3 to 13.1) | -27.9 (-30.3 to -25.7) | -15.4 (-20.7 to -9.5) | -48.1 (-51.2 to -44.6) | 9.4 (6.5 to 12.3) | -28.1 (-30.6 to -25.6) | 15.5 (12.8 to 18.4) | -3.8 (-5.9 to -1.9) | 0.1 (-6.5 to 6.8) | -21.7 (-26.8 to -16.7) | 15 (12 to 17.9) | -3.6 (-5.9 to -1.3) | 27.6 (25.2 to 29.6) | -30.7 (-32.5 to -28.5) | -15.4 (-23.3 to -6.7) | -59.4 (-63.1 to -55.3) | 25.7 (23.7 to 27.8) | -30.7 (-32.6 to -28.7) |
|  | Female | 20.1 (16.4 to 23) | -20.5 (-23.3 to -18.3) | -9.3 (-17 to 0.5) | -44.6 (-48.9 to -39.2) | 19.1 (16.1 to 22) | -20.4 (-22.8 to -18) | 18.7 (15.9 to 21.6) | -1 (-3.2 to 1.1) | 5.1 (-4.3 to 14.3) | -18.8 (-26 to -11.6) | 17.8 (14.7 to 21.1) | -0.8 (-3.3 to 1.6) | 42.5 (39.9 to 45.5) | -21.3 (-23.3 to -19.3) | -4.8 (-16.6 to 8.1) | -55 (-60.3 to -49.2) | 40.3 (37.4 to 43.4) | -21 (-23 to -19.1) |
|  | Male | 3.1 (0.1 to 6) | -33.4 (-35.8 to -31.1) | -19.7 (-26 to -9.7) | -50.9 (-54.7 to -45) | 1.9 (-1.4 to 5.2) | -33.7 (-36.3 to -31.1) | 12.8 (9.9 to 15.5) | -6.2 (-8.3 to -4.4) | -3.8 (-12.4 to 5.1) | -24.2 (-30.5 to -17.5) | 12.4 (9.6 to 15.4) | -6 (-8.3 to -3.9) | 16.3 (13.5 to 18.6) | -37.6 (-39.4 to -35.5) | -22.7 (-32.2 to -9.3) | -62.8 (-67.3 to -56.4) | 14.6 (12 to 17.1) | -37.7 (-39.7 to -35.7) |
| **SDI** | | | | | | | | | | | | | | | | | | | |
| High SDI | Both | 7 (2.1 to 12) | -20.7 (-24.6 to -17.3) | -38.2 (-41.8 to -33) | -62 (-63.8 to -59.2) | 8.5 (2.9 to 14.4) | -19.2 (-23.2 to -15.4) | 10 (6.3 to 13.9) | -2.9 (-6.2 to 0.3) | 3.6 (-0.5 to 8.4) | -18.5 (-21.6 to -14.7) | 10.5 (6.5 to 14.9) | -2 (-5.8 to 1.8) | 17.6 (14 to 21.3) | -23 (-25.8 to -20.5) | -35.9 (-40.9 to -29.8) | -69 (-70.9 to -66.6) | 20 (15.7 to 24) | -20.8 (-23.8 to -18.1) |
|  | Female | 5.4 (-0.2 to 11.5) | -19.2 (-23.3 to -15) | -36.8 (-41.8 to -29.4) | -61.4 (-63.7 to -57.4) | 7.9 (1.4 to 14.8) | -17.1 (-21.6 to -12.2) | 10.6 (6.6 to 14.8) | -0.6 (-4.2 to 2.8) | 4.1 (-1.3 to 10.4) | -17.1 (-20.9 to -12.2) | 10.9 (6.6 to 15.5) | -0.1 (-4.2 to 3.9) | 16.5 (12.3 to 21.3) | -19.7 (-22.7 to -16.5) | -34.3 (-41 to -25.7) | -68 (-70.5 to -64.6) | 19.6 (14.3 to 24.9) | -17.1 (-20.4 to -14.1) |
|  | Male | 8.5 (3.7 to 13.1) | -22.5 (-26.4 to -19.2) | -39.5 (-42.1 to -35.7) | -63.7 (-65.2 to -61.4) | 9.1 (3.9 to 14.5) | -21.4 (-25.1 to -17.9) | 9.4 (5.5 to 13.3) | -4.9 (-8.1 to -1.6) | 3.2 (-1.3 to 8.5) | -20.3 (-23.7 to -16.3) | 10.2 (6.2 to 14.6) | -3.7 (-7.4 to 0.2) | 18.7 (14.6 to 22.5) | -26.3 (-29.3 to -23.3) | -37.5 (-41.8 to -32.6) | -71.1 (-72.9 to -68.9) | 20.3 (15.9 to 24.5) | -24.3 (-27.3 to -21.4) |
| High-middle SDI | Both | 4.4 (1.1 to 7.5) | -26.7 (-29.1 to -24.6) | -13.3 (-19.7 to -7.3) | -44.9 (-48.7 to -41.3) | 5.7 (2.2 to 9.1) | -25.6 (-28.3 to -22.9) | 9.4 (5.8 to 13) | -5 (-7.3 to -2.9) | -1 (-7.7 to 5.7) | -22.3 (-27.3 to -17.2) | 8.3 (4.9 to 11.9) | -5.5 (-7.9 to -3) | 14.2 (10.9 to 17.3) | -30.4 (-32.2 to -28.5) | -14.2 (-22.4 to -6.3) | -57.2 (-61.1 to -53.5) | 14.5 (11.4 to 17.7) | -29.7 (-31.7 to -27.7) |
|  | Female | 16.3 (11.9 to 21.1) | -17.2 (-19.8 to -15) | -5 (-14.6 to 4.7) | -40.6 (-46.3 to -34.7) | 18.5 (13.9 to 23.2) | -15.3 (-18.2 to -12.7) | 11.4 (7.8 to 15.3) | -2.9 (-5.2 to -0.6) | 6.6 (-2.2 to 16.4) | -18.1 (-24.9 to -10.6) | 9.7 (5.8 to 13.7) | -3.8 (-6.6 to -0.9) | 29.5 (24.9 to 34.6) | -19.6 (-21.7 to -17.5) | 1.2 (-11 to 14.3) | -51.3 (-56.8 to -45.2) | 29.9 (24.9 to 35.5) | -18.5 (-20.9 to -16.2) |
|  | Male | -2.9 (-6.1 to 0.2) | -33.1 (-35.5 to -30.6) | -18 (-26 to -10.5) | -48.6 (-53.1 to -44) | -2.1 (-5.6 to 1.4) | -32.4 (-35.3 to -29.5) | 7.9 (4.3 to 11.6) | -6.8 (-9.1 to -4.5) | -6 (-14.7 to 2.8) | -25.5 (-32.1 to -18.9) | 7.3 (3.9 to 10.8) | -7 (-9.6 to -4.6) | 4.8 (1.9 to 7.5) | -37.6 (-39.5 to -35.3) | -22.9 (-33.6 to -12.3) | -61.7 (-66.5 to -56.9) | 5 (2.1 to 8) | -37.1 (-39.3 to -34.8) |
| Middle SDI | Both | 4.7 (1.1 to 7.7) | -37.2 (-40 to -34.6) | -8.3 (-17.2 to 1.3) | -48.2 (-53.1 to -43.1) | 10.9 (7.5 to 14.3) | -33.1 (-35.9 to -30.1) | 19.5 (15.9 to 23) | -2.4 (-4.7 to -0.3) | -1.4 (-9.3 to 7) | -26.3 (-31.8 to -20.4) | 19 (15.3 to 22.5) | -1.9 (-4.2 to 0.5) | 25.1 (21.7 to 28.4) | -38.7 (-41.1 to -36) | -9.6 (-20.4 to 3.6) | -61.9 (-66.3 to -56.5) | 32 (28.3 to 35.8) | -34.4 (-37 to -31.7) |
|  | Female | 23.6 (19.2 to 27.6) | -26.4 (-29.7 to -23.8) | -7 (-17.6 to 5.7) | -48.5 (-54 to -41.5) | 22.7 (18.8 to 26.9) | -26.3 (-29.3 to -23.3) | 22.8 (19.1 to 26.8) | -0.7 (-3.2 to 1.7) | 4 (-6.6 to 14.8) | -24.5 (-32 to -17) | 22.8 (18.6 to 26.8) | 0.6 (-2 to 3.4) | 51.8 (46.6 to 57.9) | -26.9 (-29.7 to -24.1) | -3.3 (-19.7 to 15) | -61.1 (-66.8 to -53.9) | 50.7 (45.3 to 57.6) | -25.8 (-29.1 to -22.9) |
|  | Male | -7.9 (-11.5 to -4.7) | -44.4 (-47 to -41.8) | -9.2 (-20.5 to 5.6) | -47.9 (-54.2 to -39.8) | 3 (-0.7 to 6.7) | -37.4 (-40.2 to -34.3) | 16.5 (13.2 to 19.8) | -3.9 (-6.3 to -1.8) | -5.1 (-15.3 to 6) | -27.5 (-34.5 to -19.6) | 16 (12.5 to 19.3) | -3.8 (-6.3 to -1.5) | 7.3 (4.2 to 10) | -46.5 (-48.9 to -43.8) | -13.8 (-27.5 to 5) | -62.2 (-68 to -54.8) | 19.5 (16 to 22.9) | -39.8 (-42.3 to -36.9) |
| Low-middle SDI | Both | 5.6 (2.4 to 8.6) | -36 (-38 to -34.1) | -16.6 (-24.8 to -5.9) | -49.2 (-54.1 to -43.2) | 2.1 (-1.1 to 5.1) | -37.4 (-39.5 to -35.4) | 16.3 (13.3 to 19.2) | -7.3 (-9.4 to -5.4) | -0.5 (-11.3 to 11.5) | -23.9 (-32.1 to -14.6) | 15.6 (12.3 to 18.6) | -6.9 (-9.2 to -4.7) | 22.8 (19 to 26.4) | -40.7 (-42.3 to -38.9) | -17 (-28.8 to -2.3) | -61.4 (-67.1 to -54.3) | 18 (14.3 to 21.9) | -41.7 (-43.5 to -39.8) |
|  | Female | 18 (14.9 to 21.3) | -29.7 (-31.9 to -27.7) | -4.4 (-17.7 to 13.9) | -44.7 (-51.5 to -33.9) | 13.1 (10 to 16.2) | -31 (-32.9 to -29.1) | 21.3 (17.8 to 25) | -3.8 (-6.3 to -1.4) | 4.6 (-11.1 to 22.3) | -22.6 (-34.1 to -9.9) | 20 (16.1 to 23.6) | -3.5 (-6.2 to -1) | 43.1 (38.4 to 48) | -32.4 (-34.1 to -30.7) | 0 (-18.9 to 22.7) | -57.2 (-64.7 to -46.4) | 35.7 (30.5 to 40.5) | -33.4 (-35.4 to -31.5) |
|  | Male | -4.3 (-7.7 to -1.1) | -40.7 (-42.7 to -38.7) | -24.5 (-33.4 to -7.3) | -52.1 (-57.8 to -41.5) | -7.1 (-10.8 to -3.6) | -42.2 (-44.5 to -40.1) | 11.4 (8.2 to 14.2) | -10.8 (-13.1 to -8.9) | -4.6 (-17.2 to 10.2) | -24.8 (-34.3 to -13.5) | 11.3 (8.1 to 14.1) | -10.3 (-12.5 to -8) | 6.5 (2.6 to 10.1) | -47.1 (-48.8 to -45.2) | -28 (-42 to -5.3) | -64 (-70.9 to -52.5) | 3.4 (-0.4 to 7.4) | -48.1 (-50 to -46.2) |
| Low SDI | Both | 29.4 (25.4 to 33.2) | -25.9 (-28 to -23.8) | -0.6 (-11 to 11.1) | -39 (-45 to -31.8) | 34 (29.7 to 38.4) | -22.9 (-25.2 to -20.5) | 22.8 (20 to 25.4) | -6.1 (-8.2 to -4.2) | 3.2 (-9.1 to 13.2) | -20.8 (-29.8 to -12.9) | 21.8 (18.7 to 24.8) | -6.4 (-8.4 to -4.4) | 58.9 (55 to 63.3) | -30.4 (-31.8 to -28.9) | 2.6 (-13.4 to 19.5) | -51.7 (-59.3 to -42.8) | 63.3 (58.7 to 68.1) | -27.8 (-29.5 to -26.1) |
|  | Female | 45.1 (41 to 48.9) | -17.7 (-20.2 to -15.5) | 7.7 (-8.3 to 33.7) | -36.1 (-45.5 to -20.9) | 43.4 (39.8 to 47) | -17.6 (-19.7 to -15.6) | 24.1 (20.8 to 26.9) | -5.2 (-7.4 to -3.1) | 7.4 (-6 to 21.8) | -17.9 (-28 to -5.9) | 23.2 (19.7 to 26.6) | -5.1 (-7.4 to -2.9) | 80 (75.7 to 84.2) | -21.9 (-23.6 to -20.3) | 15.7 (-6 to 54.4) | -47.6 (-57.2 to -28.6) | 76.6 (71.7 to 81.4) | -21.8 (-23.5 to -20.1) |
|  | Male | 14.1 (9.7 to 18.5) | -34.2 (-36.5 to -32.1) | -6.8 (-18 to 9.1) | -41.2 (-48.2 to -30.7) | 24.5 (18.8 to 30) | -28.3 (-31.3 to -25.4) | 21.3 (18.4 to 24.2) | -7.5 (-9.6 to -5.1) | -0.3 (-15.8 to 14.2) | -23.6 (-35.2 to -12.7) | 20.2 (16.9 to 23.4) | -7.9 (-10.1 to -5.7) | 38.4 (34 to 43.3) | -39.1 (-40.6 to -37.4) | -7.1 (-25 to 16.8) | -55 (-63.6 to -42.9) | 49.6 (43.5 to 55.5) | -34 (-36.2 to -31.9) |
| **GBD Super-region** | | | | | | | | | | | | | | | | | | | |
| Central Europe, Eastern Europe, and Central Asia | Both | 2.3 (-1 to 5.4) | -8.1 (-10.6 to -6.1) | 10.8 (7.8 to 15.4) | -6.7 (-9.2 to -3) | 2.9 (0.1 to 5.9) | -7.8 (-9.9 to -5.5) | 2.3 (-1.3 to 5.7) | -3.1 (-5.2 to -1) | -2.8 (-10.4 to 4.7) | -14 (-20.6 to -7.4) | 1 (-2 to 4.6) | -4 (-6 to -2) | 4.7 (1.8 to 7.5) | -10.9 (-12.3 to -9.3) | 7.7 (-1.3 to 17.8) | -19.8 (-26.3 to -12.5) | 3.9 (1.4 to 6.5) | -11.5 (-12.9 to -10.1) |
|  | Female | 13.1 (9.3 to 16.7) | 1.2 (-1 to 3.3) | 29 (25 to 34.2) | 6.9 (3.8 to 11.4) | 13.3 (9.9 to 17.3) | 1.5 (-1 to 4.2) | 6.3 (2.5 to 10) | 0.8 (-1.4 to 2.9) | 6.3 (-3.5 to 17.5) | -8.2 (-16.6 to 1.6) | 5.2 (1.6 to 8.8) | 0.2 (-2.3 to 2.5) | 20.2 (16.3 to 23.7) | 2 (0.5 to 3.6) | 37.1 (23.5 to 51.8) | -1.9 (-11.5 to 8.7) | 19.2 (15.5 to 22.7) | 1.7 (0.2 to 3.2) |
|  | Male | -4 (-7.4 to -0.9) | -14.5 (-17.4 to -12) | 1.9 (-1.6 to 7.6) | -16.5 (-19.2 to -11.9) | -3.3 (-6.2 to -0.3) | -14.2 (-16.5 to -11.9) | -0.5 (-4 to 3.1) | -5.9 (-8 to -3.6) | -8.6 (-18.7 to 1.5) | -18.5 (-27.1 to -9.9) | -2 (-5.1 to 1.6) | -7.2 (-9.3 to -4.9) | -4.6 (-7.3 to -1.7) | -19.6 (-21.3 to -17.5) | -6.9 (-17.4 to 4.6) | -31.9 (-39.4 to -23.7) | -5.3 (-7.4 to -2.5) | -20.3 (-22.1 to -18.5) |
| High-income | Both | 5.5 (0.5 to 10.7) | -20.6 (-24.5 to -17.2) | -41.3 (-44.9 to -36) | -64 (-65.7 to -61.1) | 7.3 (1.6 to 13.5) | -18.8 (-22.8 to -14.6) | 8.1 (4.5 to 12.1) | -3 (-6.3 to 0.1) | 4.5 (0.4 to 9.1) | -17 (-20.1 to -13.5) | 8.8 (4.9 to 13.3) | -2 (-5.9 to 1.8) | 14.1 (10.6 to 17.9) | -23 (-25.5 to -20.4) | -38.7 (-43.6 to -32.4) | -70.1 (-72 to -67.5) | 16.8 (12.3 to 21) | -20.4 (-23.3 to -17.8) |
|  | Female | 3.9 (-1.5 to 10) | -19.6 (-23.7 to -15.4) | -39.2 (-44.2 to -31.7) | -63.2 (-65.5 to -59.3) | 6.5 (0.1 to 13.6) | -17.3 (-22 to -12.3) | 9.2 (5.3 to 13.4) | -0.6 (-4.1 to 2.9) | 5 (0.1 to 11.1) | -15.6 (-19.2 to -11) | 9.5 (5.2 to 14.2) | 0 (-4.1 to 3.9) | 13.5 (9.4 to 18.2) | -20 (-23.1 to -17) | -36.2 (-42.6 to -27.5) | -69 (-71.3 to -65.6) | 16.7 (11.4 to 22.1) | -17.4 (-20.6 to -14.1) |
|  | Male | 7.1 (2.2 to 11.7) | -21.8 (-25.7 to -18.5) | -43.4 (-46 to -39.6) | -65.8 (-67.2 to -63.4) | 8.1 (2.6 to 13.8) | -20.3 (-24.3 to -16.6) | 7.2 (3.3 to 11.2) | -5.1 (-8.3 to -1.8) | 4 (-0.3 to 8.9) | -18.8 (-22 to -15.2) | 8.2 (4.1 to 12.6) | -3.7 (-7.4 to 0.4) | 14.8 (10.7 to 18.5) | -25.8 (-28.4 to -23.1) | -41.1 (-45.4 to -36.1) | -72.2 (-74.1 to -69.9) | 16.9 (12.4 to 21.3) | -23.2 (-26.2 to -20.2) |
| Latin America and Caribbean | Both | -1.3 (-4.4 to 2.3) | -45.7 (-47.7 to -43.8) | -6.1 (-10.6 to 1.3) | -53.7 (-55.8 to -49.7) | -4.3 (-7.4 to -0.8) | -46.9 (-48.9 to -44.8) | 2.6 (-0.8 to 6) | -18.8 (-21.3 to -16.4) | 12.7 (4.3 to 23.4) | -16.9 (-23 to -9.3) | -0.6 (-4.4 to 3.3) | -20.7 (-23.7 to -17.7) | 1.3 (-2.2 to 5.2) | -55.9 (-57.4 to -54.6) | 5.9 (-4 to 17.8) | -61.5 (-65 to -57) | -4.9 (-8.1 to -1.3) | -57.9 (-59.4 to -56.5) |
|  | Female | 5.4 (1.4 to 9.7) | -43.4 (-45.6 to -41.3) | 1.5 (-4.1 to 11.6) | -53.2 (-55.6 to -48.3) | 2.4 (-1.2 to 6.9) | -44.2 (-46.3 to -42) | 4.6 (0.9 to 8.3) | -18.3 (-21.1 to -15.8) | 16.5 (7 to 28.1) | -15.8 (-22.7 to -7.4) | 1.4 (-2.8 to 6) | -19.9 (-23 to -16.7) | 10.3 (5.6 to 16) | -53.7 (-55.2 to -52.1) | 18.3 (6.6 to 33.3) | -60.6 (-64.2 to -55.5) | 3.8 (-0.8 to 8.9) | -55.3 (-56.9 to -53.7) |
|  | Male | -6 (-8.9 to -2.7) | -47.2 (-49.2 to -45.2) | -11.3 (-16.2 to -3.9) | -53.5 (-55.9 to -49.6) | -9.1 (-12.3 to -5.8) | -48.6 (-50.6 to -46.6) | 0.9 (-2.5 to 4.5) | -19 (-21.7 to -16.4) | 9.8 (-0.1 to 21.5) | -17.5 (-24.7 to -8.6) | -2.2 (-5.9 to 1.8) | -21.4 (-24.2 to -18.3) | -5.1 (-8.1 to -2.1) | -57.3 (-58.9 to -55.8) | -2.6 (-12.8 to 9.1) | -61.7 (-65.6 to -57) | -11.1 (-13.9 to -8.1) | -59.6 (-61.1 to -58.1) |
| North Africa and Middle East | Both | 0.3 (-3.6 to 4) | -38.6 (-41.1 to -36.2) | -15 (-25.4 to -3.3) | -50.4 (-56.3 to -43.7) | -0.9 (-4.5 to 2.8) | -39.1 (-41.6 to -36.3) | 16 (12.3 to 20.2) | -5.1 (-7.3 to -2.9) | -6.1 (-16.1 to 5.2) | -30.8 (-37.9 to -23.2) | 16.9 (13.1 to 20.7) | -3.6 (-6 to -1.3) | 16.4 (11.8 to 20.6) | -41.7 (-43.9 to -39.7) | -20.2 (-33 to -5.2) | -65.7 (-70.9 to -60.1) | 15.8 (11.3 to 20) | -41.3 (-43.5 to -39) |
|  | Female | 9 (3.6 to 14.2) | -35.1 (-38 to -32.5) | -16 (-29.2 to 0.6) | -52.5 (-59.7 to -43.2) | 8.1 (3.1 to 12.9) | -35.3 (-38.1 to -32.6) | 18.6 (14.4 to 23.5) | -5.1 (-7.8 to -2.6) | -3.1 (-16.3 to 11.6) | -31 (-40.2 to -21.2) | 19.9 (15.5 to 24.7) | -3.3 (-6.1 to -0.3) | 29.3 (22.8 to 35.4) | -38.4 (-40.9 to -35.9) | -18.6 (-36.2 to 1.8) | -67.3 (-73.8 to -59.2) | 29.5 (22.7 to 36.6) | -37.4 (-40 to -35.1) |
|  | Male | -4.4 (-8 to -0.7) | -40.3 (-43 to -37.7) | -14.4 (-28.4 to 3.2) | -48.1 (-56.1 to -38.8) | -5.8 (-9.5 to -1.8) | -41 (-43.7 to -37.9) | 14.3 (10.7 to 18.2) | -5 (-7.3 to -2.9) | -7.9 (-21.3 to 7.5) | -30.2 (-39.4 to -19.8) | 15.1 (11.3 to 18.7) | -3.7 (-6.1 to -1.6) | 9.2 (4.9 to 13.2) | -43.3 (-45.6 to -40.8) | -21.1 (-37.6 to 0.8) | -63.8 (-70.6 to -54.7) | 8.4 (4.3 to 12.4) | -43.2 (-45.6 to -40.4) |
| South Asia | Both | 62.9 (59 to 66.2) | -8.1 (-10.1 to -6.3) | 33.5 (17.6 to 55) | -17.4 (-26.9 to -5.2) | 64.4 (60.5 to 68.2) | -7.6 (-9.6 to -5.5) | 28.5 (25.5 to 31.1) | -0.8 (-2.8 to 0.9) | 6.8 (-9.1 to 21.6) | -18 (-29.2 to -7.5) | 28.3 (25.1 to 31.4) | -0.5 (-2.6 to 1.5) | 109.4 (105.5 to 113.1) | -8.9 (-10 to -7.8) | 42.5 (16.2 to 76.3) | -32.3 (-44.5 to -19.2) | 110.8 (107.2 to 114.5) | -8 (-9.2 to -6.7) |
|  | Female | 73.8 (69.9 to 77.5) | -2.2 (-4.3 to -0.1) | 46.9 (25.9 to 73.6) | -9.5 (-22.2 to 6.9) | 75.4 (71.4 to 79.4) | -1.5 (-3.6 to 0.7) | 28.8 (25.6 to 31.8) | -0.6 (-2.6 to 1.4) | 9.8 (-7 to 29.5) | -15.9 (-27.8 to -0.7) | 28.7 (25.2 to 32.4) | -0.1 (-2.4 to 2.2) | 123.8 (119.3 to 127.8) | -2.8 (-4.1 to -1.5) | 61.2 (29.2 to 107.4) | -23.9 (-38 to -3.5) | 125.7 (121.8 to 130) | -1.5 (-2.9 to -0.2) |
|  | Male | 51.6 (47.3 to 55.6) | -14 (-16 to -11.9) | 25 (4.7 to 52.4) | -22.6 (-34.5 to -7.5) | 52.5 (48.3 to 56.8) | -13.6 (-15.7 to -11.3) | 28.2 (25.2 to 31.1) | -1.1 (-3.1 to 0.9) | 4.5 (-15.4 to 26) | -19.3 (-34.2 to -3.9) | 27.7 (24.6 to 30.7) | -0.9 (-3 to 1) | 94.4 (90 to 98.7) | -14.9 (-16.3 to -13.6) | 30.6 (-1.6 to 76.6) | -37.5 (-52.3 to -18.5) | 94.7 (90.2 to 99) | -14.4 (-16 to -12.9) |
| Southeast Asia, East Asia, and Oceania | Both | -6.3 (-10.5 to -2.3) | -42.7 (-45 to -40.2) | -18 (-30.8 to -2) | -52.3 (-59.4 to -43.8) | -7 (-11.1 to -2.7) | -43 (-45.6 to -40.1) | 13.6 (9.3 to 18.4) | -7.4 (-9.8 to -5.2) | -9.8 (-22.7 to 4.7) | -34.3 (-43.1 to -24.8) | 14.9 (10.5 to 19.3) | -5.8 (-8.2 to -3.4) | 6.4 (1.3 to 11.3) | -46.9 (-48.8 to -45.1) | -26.1 (-40.9 to -8.2) | -68.7 (-74.6 to -61.8) | 6.8 (1.5 to 11.8) | -46.3 (-48.3 to -44.4) |
|  | Female | 1.1 (-4.5 to 6.7) | -40.2 (-42.9 to -37.5) | -19.1 (-36.8 to 3.3) | -54.3 (-64 to -41.3) | 0.5 (-5.1 to 6.3) | -40.4 (-43 to -37.7) | 16.6 (11.7 to 22.1) | -7.9 (-10.6 to -5.1) | -7.5 (-24.8 to 11.7) | -35.3 (-47 to -22.2) | 18.3 (13.4 to 23.5) | -5.9 (-9 to -2.6) | 17.8 (10.6 to 24.5) | -44.9 (-47.1 to -42.5) | -25.2 (-47.7 to 2.1) | -70.4 (-78.9 to -59.6) | 18.8 (11 to 26.5) | -43.9 (-46.3 to -41.4) |
|  | Male | -10.3 (-14 to -5.9) | -43.6 (-46.2 to -40.7) | -17.4 (-34.3 to 4.8) | -49.9 (-59.4 to -38) | -10.9 (-15.1 to -6.3) | -44.2 (-47.1 to -40.9) | 11.8 (7.6 to 16.6) | -6.9 (-9.3 to -4.7) | -11.2 (-28.2 to 9.1) | -33.3 (-44.5 to -20.2) | 12.8 (8.7 to 16.9) | -5.5 (-8 to -3.3) | 0.4 (-4.5 to 5.1) | -47.5 (-49.6 to -45.2) | -26.6 (-45.1 to -0.3) | -66.6 (-74.2 to -56.1) | 0.6 (-4.4 to 5.5) | -47.3 (-49.6 to -44.6) |
| Sub-Saharan Africa | Both | 28.9 (25.1 to 32.8) | -21.2 (-23.4 to -19.1) | -7 (-16.5 to 5.7) | -45.2 (-51.1 to -36.7) | 24.3 (21.1 to 27.3) | -22.9 (-24.7 to -21.1) | 23.5 (20.2 to 27.1) | 0.3 (-2.5 to 2.9) | 3 (-7.4 to 13.4) | -21.5 (-28.9 to -14.2) | 23.4 (19.8 to 27.4) | 1.4 (-1.4 to 4.2) | 59.2 (53 to 65.9) | -21 (-23.6 to -18.2) | -4.2 (-18.3 to 15.2) | -57 (-63.4 to -47.7) | 53.4 (46.6 to 60.5) | -21.8 (-24.6 to -19.2) |
|  | Female | 38.8 (34.1 to 43.5) | -17 (-20 to -14.4) | -8.8 (-19.4 to 6.5) | -48.1 (-53.9 to -39.2) | 35.9 (32.1 to 39.8) | -17.6 (-20 to -15.1) | 24.3 (20.3 to 28.5) | 0.1 (-2.7 to 3) | 6.8 (-6.1 to 20.1) | -20.3 (-29.6 to -10.8) | 24.2 (20.1 to 28.9) | 1.3 (-1.7 to 4.3) | 72.5 (65.1 to 80.3) | -16.9 (-20.3 to -13.8) | -2.6 (-18.4 to 18.7) | -58.7 (-65.2 to -49.6) | 68.8 (61.5 to 77) | -16.5 (-19.7 to -13.3) |
|  | Male | 22.4 (18.6 to 26.5) | -24.4 (-26.6 to -22.1) | -5.7 (-17.6 to 11) | -42.4 (-49.1 to -31.9) | 16.8 (13.5 to 20) | -26.8 (-28.7 to -25) | 22.9 (19.4 to 26.6) | 0.2 (-2.7 to 2.8) | 0.4 (-12.7 to 15.6) | -22.3 (-31.7 to -12) | 22.9 (18.9 to 26.7) | 1.4 (-1.6 to 4.3) | 50.4 (44.3 to 56.8) | -24.3 (-26.8 to -21.4) | -5.3 (-21 to 19.9) | -55.2 (-62.4 to -43) | 43.5 (36.7 to 50.1) | -25.7 (-28.6 to -23) |
| **GBD regions** | | | | | | | | | | | | | | | | | | | |
| East Asia | Both | 48.5 (42.1 to 54.4) | -14.4 (-17.6 to -11) | 28.3 (7.8 to 55) | -27.2 (-38.2 to -13.8) | 45.8 (39.6 to 51.8) | -15.5 (-18.6 to -12.4) | 22 (15.6 to 27.2) | -3.5 (-7.7 to -0.2) | 13.5 (-1.7 to 32.7) | -13.5 (-23.9 to -0.3) | 19.1 (12.9 to 25.2) | -4.7 (-8.5 to -0.8) | 81.1 (72.4 to 89) | -17.4 (-20.4 to -14.1) | 45.6 (12.8 to 89.6) | -37 (-49.7 to -20.4) | 73.7 (64.9 to 82.9) | -19.5 (-22.5 to -16.2) |
|  | Female | 64.4 (56 to 72.6) | -8.2 (-12.4 to -3.8) | 42.1 (17 to 77.1) | -21.2 (-35.2 to -2.7) | 65.3 (57.9 to 72.7) | -8.2 (-12.1 to -4.2) | 30.1 (23.5 to 37.3) | 0.9 (-3.8 to 5.6) | 14.4 (-3.5 to 37.3) | -12.1 (-25 to 3.1) | 27.7 (21 to 34.7) | -0.1 (-4.9 to 4.4) | 113.8 (102.8 to 124) | -7.3 (-11 to -3.2) | 62.6 (27.8 to 113.6) | -30.7 (-45.3 to -11.6) | 111.1 (100.3 to 121.9) | -8.3 (-11.9 to -4.4) |
|  | Male | 42.5 (35.6 to 49.6) | -17.3 (-20.8 to -13) | 21.2 (-4.6 to 57.2) | -30.3 (-43.3 to -14.2) | 39 (31.7 to 46.2) | -18.7 (-22.3 to -14.8) | 18.5 (11 to 25.2) | -5.6 (-10.7 to -1.2) | 12.9 (-4.4 to 35.1) | -14.6 (-25.5 to -0.6) | 15.6 (7.8 to 23.4) | -6.6 (-11.4 to -1.4) | 68.8 (57.4 to 78.9) | -21.9 (-25.8 to -17.9) | 36.8 (2.9 to 84.3) | -40.4 (-52.9 to -23.5) | 60.6 (50.5 to 71.3) | -24.1 (-27.8 to -19.9) |
| Southeast Asia | Both | 8.6 (2 to 15.8) | -19.3 (-23.9 to -13.3) | 28.5 (20.8 to 38.9) | 5.4 (-1.3 to 14.7) | 6.7 (1.8 to 11.6) | -20.7 (-23.9 to -17.2) | 12 (7 to 17.2) | -5.8 (-9.7 to -2.2) | -2.9 (-12.8 to 8.2) | -19.4 (-27 to -11.2) | 10.3 (5.9 to 14.8) | -7.4 (-10.4 to -4.4) | 21.6 (16 to 28.3) | -24 (-28 to -19.1) | 24.8 (10.3 to 41.7) | -15.1 (-24.2 to -2.9) | 17.8 (12.7 to 22.6) | -26.6 (-29.6 to -23.4) |
|  | Female | 22.5 (15.6 to 29.4) | -6 (-10.2 to -0.9) | 52.1 (40.6 to 70.6) | 31.5 (21.2 to 50.8) | 22.5 (16.6 to 28.1) | -6.3 (-10 to -2.3) | 12.7 (7.1 to 19.1) | -5.7 (-9.9 to -0.9) | -0.2 (-11.8 to 13) | -15.1 (-24.4 to -4.6) | 11.5 (6.4 to 17.3) | -6.8 (-10.3 to -3) | 38 (30.9 to 45.6) | -11.4 (-15.4 to -6.8) | 51.8 (32.3 to 76.8) | 11.6 (-2.4 to 30.2) | 36.6 (30.1 to 43.6) | -12.6 (-16.3 to -9.3) |
|  | Male | 2.6 (-4.7 to 11.1) | -26.3 (-31.5 to -19.2) | 19 (10.6 to 29.3) | -8.2 (-14.4 to 0.6) | 0.3 (-5.3 to 5.9) | -27.8 (-31.5 to -23.7) | 11.7 (5.4 to 18.5) | -6.1 (-10.9 to -0.8) | -4.2 (-16.4 to 10.3) | -21.9 (-30.7 to -11.6) | 9.7 (4.5 to 15.5) | -8 (-11.6 to -4.1) | 14.6 (7.4 to 22.9) | -30.7 (-35.6 to -25.4) | 14 (-1.5 to 32.4) | -28.3 (-37.1 to -16.3) | 10 (3.9 to 16.5) | -33.6 (-37.2 to -29.7) |
| Oceania | Both | 8.6 (5.1 to 12.2) | -6 (-8.5 to -3.5) | -14.6 (-18.2 to -9) | -35.6 (-38.2 to -31.8) | 10.1 (7.1 to 13) | -5.1 (-7 to -2.9) | 0.1 (-3.7 to 4.1) | -4.5 (-7 to -1.7) | -3.3 (-14.2 to 8.3) | -17.6 (-26.9 to -7.7) | -1.8 (-4.3 to 1) | -5.9 (-7.8 to -4) | 8.7 (4.7 to 12.8) | -10.3 (-12.9 to -7.6) | -17.4 (-27.4 to -6.4) | -47 (-53.2 to -40) | 8.1 (4.5 to 11.5) | -10.7 (-13 to -8.5) |
|  | Female | 18.7 (14 to 23.2) | 1.2 (-1.7 to 4.3) | 3.1 (-2.4 to 9.9) | -27.4 (-30.9 to -22.9) | 19.3 (15.2 to 23.1) | 1.6 (-1.1 to 4.2) | 0.8 (-3.4 to 5.3) | -2.9 (-5.7 to 0.2) | 0.2 (-12.1 to 12.5) | -17 (-27.1 to -6.5) | 0.1 (-2.7 to 3.1) | -3.2 (-5.4 to -0.8) | 19.7 (14.9 to 24.5) | -1.7 (-4.7 to 1.1) | 3.3 (-9.8 to 16.7) | -39.7 (-47.1 to -31.8) | 19.3 (14.6 to 24.1) | -1.7 (-4.5 to 1.2) |
|  | Male | 1.1 (-3.1 to 5.4) | -11.9 (-14.9 to -8.9) | -25.2 (-28.9 to -18.1) | -41 (-43.9 to -35.3) | 3.2 (0 to 6.3) | -10.6 (-13 to -8.2) | -0.5 (-4.6 to 4) | -5.8 (-8.9 to -2.4) | -6.2 (-19.3 to 8.5) | -18.8 (-29.9 to -6.2) | -3.4 (-6.5 to -0.2) | -8.1 (-10.5 to -5.7) | 0.6 (-3.6 to 5.1) | -17 (-20.2 to -14) | -29.8 (-39.7 to -18.5) | -52.1 (-58.9 to -44.6) | -0.3 (-4 to 3.1) | -17.8 (-20.5 to -15.2) |
| Central Asia | Both | -1.1 (-4.7 to 2.6) | -6 (-8.7 to -3.5) | 26.2 (20.7 to 33.8) | 12.6 (7.9 to 19.2) | -0.4 (-3.5 to 3.3) | -5.6 (-8.4 to -2.7) | 1.4 (-2.8 to 6.1) | -0.5 (-2.9 to 1.9) | -2.6 (-13 to 8.7) | -11.4 (-20.9 to -0.9) | 0.5 (-3.7 to 5.3) | -1.1 (-3.9 to 1.7) | 0.4 (-3.4 to 4.1) | -6.5 (-8.2 to -4.8) | 22.9 (9.4 to 39.3) | -0.2 (-11.1 to 13.1) | 0.1 (-3.1 to 3.6) | -6.6 (-8.5 to -4.7) |
|  | Female | 9.4 (5.1 to 13.6) | 3.3 (0.8 to 6.1) | 47.8 (41.6 to 56.4) | 30.3 (24.8 to 38.8) | 9.6 (5.8 to 13.9) | 3.6 (0.7 to 6.8) | 7.9 (3.4 to 12.5) | 5.8 (2.9 to 8) | 11.4 (-3.5 to 27.6) | -2.1 (-15.3 to 12.6) | 6.7 (2 to 11.3) | 5.4 (2.2 to 8.4) | 18 (13.4 to 22.3) | 9.3 (7.5 to 11.2) | 64.7 (41.6 to 88.6) | 27.6 (9.5 to 46.8) | 16.9 (12.8 to 20.9) | 9.2 (7.2 to 11.1) |
|  | Male | -7 (-10.5 to -3.4) | -12.3 (-15.3 to -9.7) | 16.4 (10.1 to 25.1) | 0 (-5.1 to 6.4) | -6.1 (-9.4 to -2.4) | -12 (-15 to -9) | -2.9 (-7.1 to 1.9) | -4.9 (-7.4 to -2.2) | -10.6 (-24.2 to 3.6) | -17.1 (-29.4 to -4.3) | -3.7 (-7.8 to 1.2) | -5.6 (-8.4 to -2.7) | -9.7 (-13.1 to -6.1) | -16.6 (-18.5 to -14.8) | 4.1 (-11.6 to 22.2) | -17.1 (-29.7 to -3.3) | -9.6 (-12.6 to -5.7) | -16.9 (-18.9 to -14.6) |
| Central Europe | Both | 24.4 (18.9 to 29.9) | -10.6 (-14.9 to -6.8) | -27.5 (-36.8 to -19.3) | -69.3 (-72.5 to -66.5) | 26 (19.8 to 32.2) | -7.7 (-12.1 to -3.5) | 7.3 (3.4 to 11.6) | -0.9 (-4.1 to 2.6) | 7.8 (1.3 to 13.6) | -24.7 (-28 to -21) | 8.6 (4 to 13.7) | 1.2 (-3.1 to 5.6) | 33.5 (26.8 to 40.4) | -11.4 (-15.9 to -6.5) | -21.9 (-33.7 to -11.3) | -76.9 (-79.3 to -74.7) | 36.8 (29.4 to 43.9) | -6.6 (-11.3 to -1.5) |
|  | Female | 24.6 (18.6 to 31.7) | -17.9 (-22.3 to -13.1) | -24.8 (-38.7 to -12.3) | -72.8 (-77 to -69.3) | 27.9 (21 to 36) | -14.3 (-19.2 to -9.2) | 11.4 (6.9 to 15.7) | -0.4 (-4 to 3.6) | 17.1 (9.4 to 26.2) | -20.5 (-25.2 to -14.4) | 12.1 (7.5 to 17.2) | 1.4 (-3.2 to 6) | 38.7 (31.3 to 46.2) | -18.3 (-22.7 to -13) | -11.9 (-29.1 to 6.3) | -78.4 (-81.8 to -75.3) | 43.3 (34.8 to 51.9) | -13.1 (-18.5 to -6.8) |
|  | Male | 24.4 (18.4 to 29.8) | -7.6 (-12.4 to -3.7) | -29.9 (-38.9 to -23.6) | -66.7 (-70.5 to -63.6) | 25.1 (17.9 to 31.3) | -5.9 (-10.8 to -1) | 4.9 (0.6 to 9.4) | -2 (-5.6 to 2.2) | -0.9 (-6.3 to 4.4) | -28.9 (-32.2 to -25.4) | 6.7 (1.8 to 12.4) | 0.3 (-4.2 to 5.2) | 30.5 (23.1 to 37.9) | -9.5 (-14.5 to -3.9) | -30.6 (-39.2 to -22.4) | -76.3 (-79.1 to -73.8) | 33.4 (25.6 to 41.7) | -5.6 (-11 to 0.2) |
| Eastern Europe | Both | -14.4 (-21.6 to -6.3) | -43.5 (-48.5 to -38) | -57.1 (-61.9 to -50.7) | -77.8 (-80.2 to -74.7) | -14.8 (-21 to -7.8) | -42.9 (-47.3 to -38.5) | 14.5 (8.9 to 20.6) | -3 (-7.5 to 1.6) | 11.1 (-4.6 to 29.2) | -16.2 (-27.7 to -2.6) | 15.8 (9.8 to 22.2) | -1 (-6.2 to 3.8) | -1.9 (-12.1 to 8.4) | -45.2 (-51.3 to -38.8) | -52.4 (-59.9 to -44.1) | -81.4 (-84.2 to -78.3) | -1.3 (-10.2 to 8.2) | -43.5 (-48.9 to -37.6) |
|  | Female | -15.6 (-24.6 to -5.5) | -42.8 (-48.7 to -36.5) | -56.5 (-62.5 to -49.1) | -77.8 (-80.5 to -74.1) | -16.1 (-23.8 to -8.3) | -42 (-47.2 to -37.1) | 15.4 (8.1 to 23.9) | -1.6 (-6.9 to 4.4) | 8.5 (-7.8 to 25.8) | -15.4 (-27.5 to -2.2) | 16.5 (9.1 to 24.8) | 0.1 (-6.1 to 6.4) | -2.6 (-13.6 to 9.1) | -43.7 (-50.6 to -37.3) | -52.9 (-61 to -43.5) | -81.2 (-84.2 to -77.7) | -2.2 (-12.7 to 8.7) | -42 (-48.2 to -35.9) |
|  | Male | -13.2 (-21 to -2.8) | -44.5 (-49.7 to -37.5) | -57.7 (-62.6 to -51.4) | -78.5 (-80.8 to -75.4) | -13.6 (-20.9 to -5.7) | -43.9 (-48.4 to -38.8) | 13.7 (6 to 21.8) | -4.1 (-10.3 to 2.7) | 14 (-4.2 to 35.9) | -17.5 (-30.1 to -2.1) | 15.1 (8 to 22.3) | -1.9 (-8.3 to 4.1) | -1.3 (-12.1 to 10.5) | -46.7 (-53 to -39.4) | -51.8 (-60.3 to -43.1) | -82.2 (-85.2 to -79) | -0.6 (-10 to 10.4) | -44.9 (-50.7 to -38.8) |
| High-income Asia Pacific | Both | -8.3 (-11.6 to -4.7) | -25.6 (-28.4 to -22.9) | -43.3 (-46.9 to -36.1) | -62.2 (-64.2 to -57.8) | -5.7 (-9 to -2.4) | -22.5 (-25.2 to -19.9) | -4.4 (-8.1 to -0.7) | -10.2 (-14.2 to -6.4) | -1.1 (-6 to 4.6) | -19.5 (-23.3 to -14.9) | -5.3 (-10.1 to -0.8) | -10 (-14.7 to -5.6) | -12.3 (-16.5 to -7.8) | -33.2 (-37.2 to -29.1) | -44 (-48.9 to -35.8) | -69.6 (-71.8 to -65.4) | -10.7 (-15.4 to -5.5) | -30.2 (-34.5 to -26) |
|  | Female | -9.2 (-13 to -4.9) | -23.3 (-26.3 to -20.2) | -40.2 (-44.8 to -31.7) | -60 (-62.5 to -54.7) | -7.1 (-11.1 to -3) | -20.6 (-23.4 to -17.8) | -4.7 (-8.6 to -0.1) | -8.8 (-13 to -4.6) | -2.3 (-8.1 to 4.4) | -19.2 (-23.4 to -14.1) | -5.5 (-10.5 to -0.7) | -8.7 (-13.8 to -4) | -13.4 (-18.2 to -8.2) | -30.1 (-34.3 to -25.7) | -41.6 (-47.1 to -32.5) | -67.7 (-70.2 to -63.1) | -12.2 (-17.9 to -6) | -27.5 (-32.2 to -22.9) |
|  | Male | -7.4 (-10.9 to -3.3) | -28.5 (-31.7 to -25.5) | -46.5 (-49.5 to -40.6) | -65.5 (-67.4 to -61.8) | -4.3 (-8 to -0.6) | -24.8 (-27.7 to -22) | -4.1 (-8.3 to -0.3) | -11.5 (-15.4 to -7.9) | 0.3 (-5.8 to 7.3) | -20.3 (-25 to -15.2) | -5.1 (-9.5 to -0.6) | -11.2 (-16 to -6.8) | -11.2 (-15.4 to -6.5) | -36.7 (-40.7 to -32.7) | -46.3 (-51.3 to -39.1) | -72.5 (-74.9 to -68.6) | -9.3 (-13.8 to -4.4) | -33.2 (-37.4 to -29.2) |
| Australasia | Both | 21.7 (15.8 to 28.4) | -14.3 (-18.2 to -9.9) | -17.4 (-26 to -7.8) | -48.9 (-54.1 to -43) | 21.2 (16.4 to 26.3) | -13.7 (-17.1 to -10.1) | 13.7 (7.6 to 20.6) | -2.7 (-8 to 2.9) | 12.6 (-1.3 to 28.6) | -7.2 (-18.6 to 5.9) | 13.6 (8 to 18.9) | -2.6 (-7.6 to 2.1) | 38.3 (31.7 to 45.7) | -16.7 (-20.6 to -12.3) | -7 (-19.4 to 7.1) | -52.6 (-58.7 to -45.6) | 37.7 (30.6 to 45.1) | -15.9 (-20 to -11.5) |
|  | Female | 27 (19.1 to 35.6) | -11.9 (-16.7 to -6.3) | -4.6 (-15.3 to 7.3) | -45.1 (-50.9 to -38.6) | 26.1 (19.9 to 33) | -11.2 (-15.5 to -6.8) | 16.7 (9 to 25.1) | 0.2 (-6.1 to 6.8) | 17.1 (1.4 to 33.9) | -3.3 (-15.7 to 10.3) | 16.6 (8.9 to 23.7) | 0.4 (-6.6 to 6.3) | 48.2 (38.8 to 56.5) | -11.7 (-16.8 to -7) | 11.7 (-2.9 to 29.5) | -47 (-53.8 to -39.1) | 47 (38.7 to 55.3) | -10.9 (-15.7 to -6) |
|  | Male | 18.1 (10.3 to 27.6) | -16.3 (-22 to -9.6) | -24.8 (-33.7 to -14.7) | -51.1 (-56.9 to -44.5) | 17.9 (11.8 to 24.6) | -15.6 (-19.8 to -10.7) | 11.4 (2.8 to 20.3) | -4.9 (-12.1 to 2.5) | 9.3 (-7.4 to 28.6) | -10.2 (-23.6 to 5.1) | 11.3 (5.2 to 18) | -4.9 (-10 to 0.7) | 31.6 (22.3 to 41.4) | -20.4 (-25.8 to -14.7) | -17.9 (-31.1 to -1.6) | -56.1 (-63 to -47.3) | 31.2 (23 to 40.3) | -19.7 (-24.7 to -14.6) |
| Western Europe | Both | 5.7 (-2.6 to 15.3) | -22.1 (-27.9 to -15.7) | -48.4 (-50.6 to -45.3) | -65.8 (-67.1 to -63.9) | 6 (-3.5 to 16.8) | -22.1 (-28.8 to -14.6) | 16.4 (10.6 to 22.4) | -0.5 (-4.6 to 3.9) | 13.6 (8.9 to 19.3) | -6.2 (-10 to -1.7) | 17.2 (11.8 to 23.3) | 0.5 (-3.9 to 5) | 23 (17.2 to 30.9) | -22.5 (-26 to -18.5) | -41.4 (-44.7 to -37.5) | -68 (-69.7 to -65.9) | 24.2 (16.9 to 32.8) | -21.7 (-26 to -16.7) |
|  | Female | 5 (-3.3 to 15.1) | -18.8 (-25.1 to -11.8) | -48.2 (-51.2 to -43.2) | -64.7 (-66.4 to -61.7) | 7.5 (-2.7 to 19.1) | -17.1 (-24.8 to -8.8) | 16.1 (10.3 to 22.1) | 1.5 (-2.7 to 6.2) | 10.6 (5.1 to 18) | -6.4 (-10.7 to -0.4) | 16.1 (10.7 to 22.2) | 1.8 (-2.9 to 6.4) | 21.9 (15.9 to 30.1) | -17.6 (-21.8 to -12.6) | -42.7 (-46.7 to -37.6) | -67 (-69 to -64.4) | 24.8 (17 to 34.1) | -15.7 (-20.9 to -9.9) |
|  | Male | 6.4 (-2.2 to 15.6) | -25.3 (-30.9 to -18.9) | -48.6 (-51.2 to -46.4) | -67.6 (-69.3 to -66.2) | 4.2 (-4.7 to 14.3) | -26.9 (-32.8 to -20.2) | 16.8 (10.6 to 23.1) | -2.7 (-6.8 to 1.7) | 16.8 (11.2 to 22.8) | -7.1 (-11.5 to -2.3) | 18.5 (13 to 24.7) | -0.9 (-5 to 3.7) | 24.3 (17.9 to 32.1) | -27.3 (-30.7 to -23.6) | -40 (-43.9 to -36.1) | -70 (-71.9 to -68) | 23.4 (16.1 to 32) | -27.5 (-31.4 to -23.1) |
| Southern Latin America | Both | 17.7 (14 to 22.4) | -22.6 (-25.4 to -19.6) | -7.9 (-17.9 to 3.6) | -43.5 (-49.6 to -36.5) | 15.5 (11.9 to 19.2) | -22.8 (-25.2 to -20.4) | 15.2 (11.5 to 19.1) | -0.4 (-3.7 to 2.6) | 10.2 (-3.9 to 26.8) | -9.6 (-21.1 to 4) | 14.5 (10.9 to 17.7) | -0.2 (-3.1 to 2.5) | 35.6 (30.9 to 41) | -22.9 (-26 to -19.7) | 1.6 (-12.6 to 17.9) | -48.9 (-56.2 to -40.4) | 32.2 (27.1 to 38.2) | -23 (-26.1 to -19.8) |
|  | Female | 29.2 (23.6 to 34.9) | -15.8 (-19.4 to -11.9) | 1.2 (-11.9 to 16.8) | -40 (-47.9 to -31.3) | 27.5 (22.3 to 32.6) | -15.2 (-18.3 to -11.8) | 15.6 (11.4 to 20.1) | -0.6 (-4 to 3.1) | 9.8 (-4.1 to 25.7) | -10.2 (-21.8 to 3.1) | 14.7 (10.3 to 19.2) | -0.6 (-4.1 to 3.1) | 49.3 (42.9 to 56.9) | -16.3 (-20.2 to -12.3) | 11.1 (-7 to 32.7) | -46.2 (-54.8 to -36) | 46.2 (39.4 to 54.1) | -15.7 (-19.7 to -11.4) |
|  | Male | 11 (6.2 to 16) | -26.5 (-29.7 to -23) | -12.8 (-24.4 to 0.9) | -44.8 (-52 to -36.6) | 8.2 (4.2 to 12.4) | -27.4 (-30 to -24.5) | 14.9 (10.5 to 19.7) | -0.2 (-4.3 to 3.9) | 10.5 (-7 to 30.2) | -9 (-23 to 7.3) | 14.3 (10.4 to 18.5) | 0.1 (-3.3 to 3.6) | 27.5 (22.4 to 33.8) | -26.7 (-30 to -22.7) | -3.7 (-18.8 to 13.6) | -49.8 (-57.4 to -40.8) | 23.7 (18.7 to 29.6) | -27.3 (-30.5 to -23.9) |
| High-income North America | Both | 29.5 (23.3 to 36.4) | -29.4 (-32.7 to -25.5) | -1.4 (-14.5 to 25.2) | -49.4 (-56.1 to -34.9) | 25.2 (19.4 to 31.5) | -29.8 (-32.6 to -26.9) | 18.6 (12.7 to 23.9) | -8.8 (-13.5 to -4.7) | -1 (-18.5 to 20.1) | -27.4 (-40.2 to -12.5) | 18.6 (13.6 to 23.9) | -8.1 (-12 to -4.1) | 53.7 (45.8 to 61.8) | -35.6 (-39 to -32.1) | -2.4 (-22.7 to 28) | -63.3 (-70.7 to -51.5) | 48.5 (39.6 to 57.8) | -35.4 (-38.6 to -32.1) |
|  | Female | 39.6 (31.2 to 48.4) | -24.8 (-29.1 to -20) | 9.6 (-6.8 to 34.2) | -45.3 (-53.5 to -32.2) | 34.6 (27.4 to 43) | -24.9 (-28.2 to -20.9) | 21.6 (14.4 to 28.2) | -7.2 (-13 to -2) | 4.4 (-16.2 to 30.4) | -24.7 (-39.3 to -6.2) | 20.8 (14.8 to 27) | -7 (-11.6 to -2.2) | 69.8 (58.6 to 81.5) | -30.2 (-34 to -26.1) | 14.5 (-13.3 to 51.4) | -58.8 (-68.4 to -45.3) | 62.7 (51.8 to 75.9) | -30.1 (-33.6 to -26.3) |
|  | Male | 21.7 (15 to 29.3) | -33.3 (-37.1 to -28.6) | -9.2 (-23.4 to 19.1) | -52.4 (-59.5 to -37.7) | 17.8 (11.6 to 23.8) | -33.8 (-37 to -30.5) | 16 (9.3 to 22.9) | -10.4 (-15.8 to -5) | -5.6 (-25.3 to 17.4) | -29.9 (-44.2 to -13.1) | 16.6 (10.7 to 23.2) | -9.3 (-13.9 to -4.3) | 41.2 (31.6 to 50.7) | -40.2 (-44.3 to -36) | -14.2 (-35.2 to 21.4) | -66.7 (-74.6 to -52.9) | 37.3 (27.7 to 48) | -40 (-43.8 to -35.8) |
| Caribbean | Both | 37.6 (32.2 to 43) | -31.2 (-34.1 to -28.3) | -6.4 (-11.2 to 0) | -56.1 (-58.2 to -53) | 44.6 (39.3 to 50.4) | -24.6 (-27.6 to -21.5) | 10.2 (5.6 to 14.4) | -14.5 (-17.9 to -11.6) | 17.5 (4.6 to 33.6) | -15.5 (-24.5 to -4) | 4.5 (-0.3 to 9.4) | -17.3 (-21 to -13.9) | 51.7 (46.1 to 57.7) | -41.2 (-43 to -39.2) | 10 (-3.1 to 26.8) | -62.9 (-67.3 to -57.3) | 51.1 (44 to 58.3) | -37.7 (-39.7 to -35.7) |
|  | Female | 45.1 (38.7 to 51.7) | -31.1 (-34.6 to -27.6) | -2.3 (-8.1 to 6.4) | -56.9 (-59.4 to -52.8) | 54.5 (48 to 61.8) | -23.5 (-27.3 to -19.5) | 12 (6.6 to 17.1) | -14.7 (-18.7 to -11.2) | 21 (6.8 to 38.3) | -15 (-24.7 to -2.8) | 4 (-1.7 to 9.5) | -18.9 (-22.9 to -15.1) | 62.5 (56.1 to 69.8) | -41.2 (-43.4 to -38.7) | 18.3 (4 to 37.3) | -63.3 (-67.8 to -57.5) | 60.6 (51.8 to 69.4) | -37.9 (-40.4 to -35.4) |
|  | Male | 31.7 (26.4 to 36.7) | -30.8 (-33.6 to -28) | -9.8 (-14.9 to -3.1) | -55 (-57.5 to -51.6) | 36.5 (30.8 to 42.3) | -25.3 (-28.3 to -22.4) | 8.6 (4 to 13.3) | -14.5 (-18.1 to -11.1) | 14.3 (-1.4 to 33.6) | -15.7 (-27.1 to -1.7) | 5 (0 to 10) | -16 (-19.8 to -12.2) | 43 (37.2 to 49.5) | -40.9 (-42.9 to -38.7) | 3 (-11.9 to 21.4) | -62.1 (-67.5 to -55.6) | 43.3 (36.6 to 50.2) | -37.3 (-39.3 to -35.1) |
| Andean Latin America | Both | -21.6 (-24.2 to -18.4) | -56 (-57.6 to -54.4) | -6.7 (-12.7 to 2.9) | -54.4 (-57.2 to -49.3) | -25 (-27.9 to -21.8) | -58.1 (-60 to -56.4) | -7.9 (-12 to -3.4) | -26.7 (-29.8 to -23.4) | 11.6 (5.1 to 18.8) | -17.6 (-22.3 to -12.4) | -9.8 (-14.5 to -4.8) | -28.3 (-32 to -24.6) | -27.7 (-31.1 to -24.1) | -67.7 (-69.2 to -66.6) | 4.2 (-4.5 to 17.2) | -62.5 (-65.4 to -57.5) | -32.4 (-36 to -28.9) | -70 (-71.4 to -68.6) |
|  | Female | -17.6 (-21.1 to -13.5) | -54.3 (-56.2 to -52.4) | 5.5 (-2.7 to 21.3) | -53 (-56.4 to -45.7) | -21.9 (-25.4 to -17.7) | -56.8 (-58.6 to -54.8) | -6.2 (-10.4 to -1.1) | -26.2 (-29.5 to -22.9) | 16 (7.8 to 25) | -16.7 (-22.4 to -10.2) | -6.6 (-11.5 to -0.5) | -26.1 (-30.1 to -22) | -22.7 (-27.1 to -17.5) | -66.3 (-68 to -64.7) | 22.4 (10.1 to 43.7) | -60.8 (-64.4 to -53.8) | -27 (-31.8 to -22.2) | -68.1 (-69.9 to -66.5) |
|  | Male | -24.3 (-26.9 to -21.1) | -57.1 (-58.8 to -55.3) | -13.1 (-19.1 to -5.1) | -54.7 (-57.8 to -50) | -27.2 (-30.3 to -24.1) | -58.9 (-60.8 to -57) | -9.1 (-13.3 to -4.4) | -26.7 (-30.2 to -23.3) | 8.8 (0.6 to 17.6) | -18 (-24 to -11.5) | -12.2 (-17 to -6.9) | -29.9 (-33.7 to -26.1) | -31.2 (-34.6 to -28) | -68.6 (-70 to -67.4) | -5.5 (-14.4 to 5.2) | -62.8 (-66.4 to -58.2) | -36.1 (-39.3 to -33.1) | -71.2 (-72.6 to -69.8) |
| Central Latin America | Both | 67.6 (60.8 to 75) | -10.4 (-14 to -6.9) | 1.3 (-11.1 to 14.3) | -44.1 (-51 to -36.9) | 74.7 (67.8 to 82.1) | -6.7 (-9.9 to -3.6) | 20.3 (16.4 to 24.9) | -4.1 (-6.8 to -1.5) | 4.5 (-6 to 15.8) | -20.8 (-27.6 to -13.4) | 16.9 (12.2 to 22) | -6.1 (-9.2 to -3.1) | 101.6 (90.8 to 115.9) | -14.1 (-18.1 to -9.7) | 5.9 (-12.6 to 28.2) | -55.7 (-63.3 to -46.7) | 104.2 (91.4 to 118.7) | -12.4 (-16.4 to -8.1) |
|  | Female | 67.6 (60.8 to 75) | -10.4 (-14 to -6.9) | 1.3 (-11.1 to 14.3) | -44.1 (-51 to -36.9) | 74.7 (67.8 to 82.1) | -6.7 (-9.9 to -3.6) | 20.3 (16.4 to 24.9) | -4.1 (-6.8 to -1.5) | 4.5 (-6 to 15.8) | -20.8 (-27.6 to -13.4) | 16.9 (12.2 to 22) | -6.1 (-9.2 to -3.1) | 101.6 (90.8 to 115.9) | -14.1 (-18.1 to -9.7) | 5.9 (-12.6 to 28.2) | -55.7 (-63.3 to -46.7) | 104.2 (91.4 to 118.7) | -12.4 (-16.4 to -8.1) |
|  | Male | 77.4 (70.4 to 86) | -2.6 (-6 to 1) | 7.1 (-5.7 to 28.7) | -39.7 (-48 to -25.9) | 86.6 (77.8 to 95.8) | 2.3 (-1.2 to 6.3) | 18.9 (14.1 to 23.6) | -4.2 (-7.4 to -1) | 5.1 (-6.2 to 19.6) | -20.2 (-29 to -8.7) | 14.4 (9.3 to 19.5) | -7.2 (-10.6 to -3.7) | 110.9 (99.4 to 125.2) | -6.7 (-10.4 to -2.3) | 12.5 (-7.7 to 45.2) | -51.9 (-61.1 to -35) | 113.4 (99.7 to 128.9) | -5 (-9.2 to -0.7) |
| Tropical Latin America | Both | 77.4 (70.4 to 86) | -2.6 (-6 to 1) | 7.1 (-5.7 to 28.7) | -39.7 (-48 to -25.9) | 86.6 (77.8 to 95.8) | 2.3 (-1.2 to 6.3) | 18.9 (14.1 to 23.6) | -4.2 (-7.4 to -1) | 5.1 (-6.2 to 19.6) | -20.2 (-29 to -8.7) | 14.4 (9.3 to 19.5) | -7.2 (-10.6 to -3.7) | 110.9 (99.4 to 125.2) | -6.7 (-10.4 to -2.3) | 12.5 (-7.7 to 45.2) | -51.9 (-61.1 to -35) | 113.4 (99.7 to 128.9) | -5 (-9.2 to -0.7) |
|  | Female | 59 (50.6 to 67.6) | -17.1 (-21.3 to -13) | -2.7 (-17 to 10.8) | -47.4 (-54.8 to -40.1) | 63.8 (55.8 to 71.6) | -14.6 (-18.5 to -11) | 21.6 (17.6 to 27) | -4.1 (-6.9 to -1) | 4.1 (-8.7 to 17.8) | -21.4 (-29.9 to -12.6) | 19.5 (14.3 to 25.3) | -5 (-8.4 to -1.5) | 93.5 (81.7 to 108.6) | -20.6 (-25.3 to -15.9) | 1.2 (-22.2 to 23.9) | -58.6 (-67 to -50.1) | 95.8 (82.3 to 110.4) | -18.9 (-23.7 to -14) |
|  | Male | 59 (50.6 to 67.6) | -17.1 (-21.3 to -13) | -2.7 (-17 to 10.8) | -47.4 (-54.8 to -40.1) | 63.8 (55.8 to 71.6) | -14.6 (-18.5 to -11) | 21.6 (17.6 to 27) | -4.1 (-6.9 to -1) | 4.1 (-8.7 to 17.8) | -21.4 (-29.9 to -12.6) | 19.5 (14.3 to 25.3) | -5 (-8.4 to -1.5) | 93.5 (81.7 to 108.6) | -20.6 (-25.3 to -15.9) | 1.2 (-22.2 to 23.9) | -58.6 (-67 to -50.1) | 95.8 (82.3 to 110.4) | -18.9 (-23.7 to -14) |
| North Africa and Middle East | Both | 7.9 (4.6 to 10.9) | -37.9 (-39.8 to -36.1) | -21.5 (-30.8 to -7.6) | -55.1 (-60.4 to -47.5) | 3.2 (-0.1 to 6.3) | -39.3 (-41.2 to -37.5) | 19.2 (15.7 to 22.5) | -8.2 (-10.6 to -6.1) | -0.2 (-14.9 to 16.9) | -26.3 (-37.1 to -13.9) | 18.1 (14.4 to 21.3) | -7.1 (-9.5 to -4.8) | 28.6 (24.5 to 32.6) | -43 (-44.2 to -41.4) | -21.7 (-35.9 to -3.2) | -66.9 (-73.2 to -59.2) | 21.9 (17.6 to 26.3) | -43.6 (-45.3 to -41.8) |
|  | Female | 7.9 (4.6 to 10.9) | -37.9 (-39.8 to -36.1) | -21.5 (-30.8 to -7.6) | -55.1 (-60.4 to -47.5) | 3.2 (-0.1 to 6.3) | -39.3 (-41.2 to -37.5) | 19.2 (15.7 to 22.5) | -8.2 (-10.6 to -6.1) | -0.2 (-14.9 to 16.9) | -26.3 (-37.1 to -13.9) | 18.1 (14.4 to 21.3) | -7.1 (-9.5 to -4.8) | 28.6 (24.5 to 32.6) | -43 (-44.2 to -41.4) | -21.7 (-35.9 to -3.2) | -66.9 (-73.2 to -59.2) | 21.9 (17.6 to 26.3) | -43.6 (-45.3 to -41.8) |
|  | Male | 20 (16.5 to 23.6) | -33.3 (-35.4 to -31.5) | -7.3 (-22 to 15.4) | -51 (-58.2 to -39.6) | 14.2 (10.5 to 17.9) | -34 (-35.8 to -32.2) | 23.7 (19.9 to 27.6) | -5.5 (-8 to -3.2) | 7.1 (-13.8 to 30.5) | -24.1 (-39.1 to -7.6) | 22 (17.7 to 25.8) | -4.1 (-6.9 to -1.6) | 48.5 (42.2 to 54.7) | -36.9 (-38.4 to -35.5) | -0.7 (-23.5 to 28.8) | -62.8 (-71.7 to -50.3) | 39.4 (32.7 to 45.6) | -36.7 (-38.7 to -34.7) |
| South Asia | Both | 20 (16.5 to 23.6) | -33.3 (-35.4 to -31.5) | -7.3 (-22 to 15.4) | -51 (-58.2 to -39.6) | 14.2 (10.5 to 17.9) | -34 (-35.8 to -32.2) | 23.7 (19.9 to 27.6) | -5.5 (-8 to -3.2) | 7.1 (-13.8 to 30.5) | -24.1 (-39.1 to -7.6) | 22 (17.7 to 25.8) | -4.1 (-6.9 to -1.6) | 48.5 (42.2 to 54.7) | -36.9 (-38.4 to -35.5) | -0.7 (-23.5 to 28.8) | -62.8 (-71.7 to -50.3) | 39.4 (32.7 to 45.6) | -36.7 (-38.7 to -34.7) |
|  | Female | -2.8 (-6.3 to 1) | -42 (-44.1 to -39.9) | -31.5 (-41.9 to -9.2) | -58.5 (-64.8 to -45.5) | -7 (-11.2 to -3.2) | -44.1 (-46.4 to -41.9) | 14.3 (10.8 to 17.6) | -11.4 (-13.9 to -9.2) | -7.1 (-25.8 to 16) | -28.9 (-42.8 to -12.7) | 13.7 (10.1 to 16.9) | -10.5 (-13.2 to -8) | 11.1 (7.1 to 15.4) | -48.7 (-50.1 to -46.9) | -36.3 (-53.5 to -9.1) | -70.5 (-78.4 to -57.9) | 5.7 (1.3 to 10.1) | -50 (-51.9 to -48.1) |
|  | Male | -2.8 (-6.3 to 1) | -42 (-44.1 to -39.9) | -31.5 (-41.9 to -9.2) | -58.5 (-64.8 to -45.5) | -7 (-11.2 to -3.2) | -44.1 (-46.4 to -41.9) | 14.3 (10.8 to 17.6) | -11.4 (-13.9 to -9.2) | -7.1 (-25.8 to 16) | -28.9 (-42.8 to -12.7) | 13.7 (10.1 to 16.9) | -10.5 (-13.2 to -8) | 11.1 (7.1 to 15.4) | -48.7 (-50.1 to -46.9) | -36.3 (-53.5 to -9.1) | -70.5 (-78.4 to -57.9) | 5.7 (1.3 to 10.1) | -50 (-51.9 to -48.1) |
| Central Sub-Saharan Africa | Both | 80.1 (72.7 to 87.1) | -1.2 (-4.9 to 2.4) | 47.2 (17.2 to 85.3) | -13.3 (-29.7 to 4.8) | 80.6 (74.6 to 86.7) | -0.8 (-3.8 to 2.4) | 32.2 (26.2 to 37.7) | -1.8 (-5.8 to 2.3) | 3.8 (-14.7 to 25.7) | -19.9 (-34.1 to -5.1) | 32.1 (26.1 to 37.5) | -1 (-4.6 to 2.3) | 138.1 (129.7 to 148.3) | -3.1 (-6 to 0.1) | 52.8 (15.9 to 106.6) | -30.5 (-46.7 to -10.4) | 138.6 (129.5 to 149.2) | -1.8 (-4.9 to 1.5) |
|  | Female | 91.6 (80.9 to 102.4) | 2.3 (-2.7 to 7.6) | 89.8 (44.2 to 150.2) | -0.4 (-20.9 to 30.3) | 90.3 (82.3 to 99.3) | 2.9 (-1.1 to 7.3) | 30.8 (23.5 to 38) | -1.9 (-7.1 to 3.2) | 3.5 (-18.3 to 29.5) | -18.8 (-34.2 to 2.2) | 31.1 (24.2 to 37.8) | -1 (-5.5 to 3.5) | 150.7 (138.4 to 164.6) | 0.4 (-3.7 to 4.9) | 96.4 (38.3 to 170.2) | -19.1 (-39.2 to 11.7) | 149.4 (137.5 to 162.7) | 1.9 (-1.9 to 6.2) |
|  | Male | 68.4 (60.1 to 76.6) | -6.6 (-10.9 to -2.3) | 19.5 (-16.4 to 63.9) | -31.2 (-49.7 to -7.4) | 70.1 (62.6 to 77.3) | -6.5 (-10.2 to -2.5) | 33.7 (26.7 to 41.4) | -2 (-6.6 to 3) | 4.2 (-19.8 to 33.9) | -22.8 (-39.6 to -2) | 33.4 (26.5 to 40.6) | -1.3 (-5.5 to 3.3) | 125.1 (115.1 to 137.4) | -8.6 (-12 to -4.4) | 24.4 (-16.9 to 88.4) | -46.9 (-64.5 to -20.8) | 126.9 (115.9 to 139.7) | -7.7 (-11.4 to -3.3) |
| Eastern Sub-Saharan Africa | Both | 44.3 (40.6 to 47.8) | -17.2 (-19.2 to -15) | 7.7 (-13.1 to 27.5) | -33.3 (-46.7 to -22.9) | 43.7 (39.7 to 47.8) | -17.6 (-19.9 to -15.4) | 33.4 (30 to 36.4) | 0.5 (-1.6 to 2.5) | 11.3 (-6.4 to 26.9) | -15.4 (-27.5 to -4.9) | 33.2 (29.5 to 36.6) | 1 (-1.4 to 3.1) | 92.5 (88 to 96.8) | -16.7 (-18.1 to -15.3) | 19.8 (-17.9 to 52.1) | -43.6 (-60.4 to -30.1) | 91.4 (87.1 to 95.9) | -16.8 (-18.5 to -15.2) |
|  | Female | 41 (37 to 44.9) | -17.7 (-20 to -15.2) | 6.7 (-18.9 to 48) | -33 (-48.7 to -12.7) | 38.3 (33.7 to 43) | -19.2 (-21.5 to -16.8) | 34.9 (30.8 to 38.9) | 1.8 (-0.9 to 4.5) | 14.7 (-1.7 to 37.5) | -12.4 (-24 to 4.9) | 34.9 (30.5 to 39.5) | 2.6 (-0.2 to 5.4) | 90.2 (84.3 to 95.7) | -16.2 (-18.2 to -14.2) | 22.3 (-13.3 to 86.4) | -41.3 (-56.9 to -13) | 86.6 (80.4 to 92.8) | -17.1 (-19.3 to -14.7) |
|  | Male | 47.1 (42.2 to 51.7) | -17.2 (-19.8 to -14.5) | 8.2 (-13.2 to 29.8) | -33 (-46.3 to -20.5) | 48.2 (43.3 to 53.4) | -16.7 (-19.3 to -14) | 32.1 (28.5 to 35.5) | -0.7 (-3 to 1.5) | 9.7 (-11 to 33) | -17.2 (-32.4 to -0.9) | 31.8 (28.2 to 35.2) | -0.4 (-2.6 to 1.9) | 94.4 (89.1 to 99.9) | -17.8 (-19.7 to -15.9) | 18.6 (-20.7 to 65) | -44.5 (-63 to -23.9) | 95.3 (89.7 to 100.7) | -17.1 (-19.1 to -15.1) |
| Southern Sub-Saharan Africa | Both | 52.8 (49 to 56.9) | 0 (-2.5 to 2.2) | 85.2 (34.2 to 172.3) | 20.3 (-11.7 to 74.7) | 54.2 (50.2 to 58.6) | 0.3 (-2.2 to 2.8) | 14.6 (11 to 18.5) | -2.2 (-4.8 to 0.3) | -8.8 (-19.1 to 0.3) | -25.7 (-33 to -19.1) | 13.9 (9.7 to 17.8) | -2.3 (-5.2 to 0.5) | 75.2 (69 to 82) | -2.2 (-4.3 to -0.1) | 68.9 (23.2 to 138.7) | -10.6 (-33.3 to 25.1) | 75.7 (69.6 to 82.2) | -2 (-4.2 to 0.4) |
|  | Female | 67.1 (62 to 72.3) | 9.8 (7.5 to 12.7) | 112.3 (60.7 to 218) | 34 (0.9 to 99.7) | 67.9 (62.1 to 74.2) | 10.5 (7.6 to 13.8) | 13.7 (9.2 to 18.3) | -1.6 (-4.6 to 1.1) | -10 (-20.7 to 3.2) | -26 (-34.2 to -15.5) | 12.4 (7.6 to 17.4) | -2.2 (-5.8 to 1.4) | 90 (80.2 to 99.6) | 8 (5.9 to 10.4) | 91 (53.4 to 177.5) | -0.8 (-19.6 to 42.3) | 88.8 (79 to 98.9) | 8 (5.2 to 10.9) |
|  | Male | 40.6 (35.8 to 45) | -8.3 (-11.4 to -5.8) | 66.6 (16.6 to 162.1) | 10.8 (-20.5 to 69.8) | 42.4 (38.1 to 46.9) | -8 (-10.9 to -5.4) | 15.6 (11.5 to 20) | -3.4 (-6.4 to -0.3) | -7.7 (-21.1 to 6.3) | -26.3 (-35.7 to -17.1) | 15.4 (11.1 to 19.5) | -3.1 (-6.1 to 0.1) | 62.5 (57 to 67.9) | -11.4 (-13.9 to -8.9) | 53.8 (0.3 to 130.8) | -18.3 (-45.2 to 20.8) | 64.4 (58.7 to 69.7) | -10.8 (-13.6 to -8.2) |
| Western Sub-Saharan Africa | Both | 75.5 (70.3 to 79.7) | -5.4 (-7.7 to -3.1) | 40 (15.5 to 72.1) | -14.9 (-28.9 to 4.1) | 78.6 (74.4 to 82.6) | -3.9 (-6 to -1.8) | 26.7 (23.3 to 30.1) | -2.2 (-4.4 to 0.1) | 8.6 (-10.1 to 30.1) | -16.3 (-29.6 to -0.7) | 26.5 (22.9 to 30) | -2.2 (-4.4 to -0.1) | 122.4 (117.1 to 127.2) | -7.5 (-8.7 to -6.4) | 52 (18.1 to 94.8) | -28.7 (-44.4 to -10.9) | 125.8 (121.2 to 130.7) | -6.1 (-7.2 to -4.9) |
|  | Female | 91.8 (86.9 to 96.8) | 4.1 (1.7 to 6.4) | 48.8 (18.1 to 86.6) | -6.3 (-25.8 to 17.8) | 95.9 (91.4 to 100.5) | 6 (3.8 to 8.1) | 27.4 (23.2 to 31.3) | -2.9 (-5.1 to -0.5) | 14.3 (-7.7 to 45.1) | -13.6 (-29.5 to 8.1) | 27.5 (23.2 to 31.8) | -2.8 (-5.4 to -0.3) | 144.3 (136.9 to 150.1) | 1 (-0.7 to 2.5) | 70.1 (27.5 to 134.1) | -19.1 (-37.8 to 10.2) | 149.7 (142.9 to 156.1) | 3 (1.2 to 4.6) |
|  | Male | 54.2 (48.2 to 59.5) | -14.7 (-17.2 to -12.3) | 33.3 (2.5 to 76.1) | -20.1 (-37.3 to 4.2) | 54.8 (50 to 59.7) | -14.2 (-16.5 to -11.7) | 25.6 (21.9 to 29.2) | -1.8 (-4.3 to 0.8) | 3.8 (-20.6 to 35) | -17.6 (-36.6 to 6.1) | 24.7 (21 to 28.1) | -2.1 (-4.4 to 0.2) | 93.6 (87.5 to 99.5) | -16.3 (-17.8 to -14.8) | 38.3 (-4.3 to 101.4) | -34.2 (-54 to -7.2) | 93 (86.6 to 98.5) | -15.9 (-17.6 to -14.4) |

Data in parenthesis are 95% uncertainty intervals (95% UI)
